# Scenario-Driven Synthetic Data Generation Framework for Visual Perception Evaluation Under Adverse Driving Conditions

**DOI:** 10.3390/s26113464

**Published:** 2026-05-30

**Authors:** Wei Xu, Dominique Gruyer, Alexandra Duminil, Sio-Song Ieng

**Affiliations:** 1 Perceptions, Interactions, Behaviour and Simulations of Road and Street Users Laboratory (PICS-L), Department of Components and Systems (COSYS), Gustave Eiffel University, 77454 Marne-la-Vallée, France; dominique.gruyer@univ-eiffel.fr (D.G.); alex.duminil@orange.fr (A.D.); sio-song.ieng@univ-eiffel.fr (S.-S.I.); 2 Doctoral School of Information and Communication Sciences and Technologies (ED STIC), Paris-Saclay University, 91190 Gif-sur-Yvette, France

**Keywords:** synthetic datasets, autonomous driving, ODD and OEDR, adverse conditions, visual perception evaluation, scenario-based framework

## Abstract

Developing and evaluating visual perception systems for autonomous vehicles requires data across diverse and adverse driving conditions, yet collecting and annotating such real-world data is costly and often impractical. To address this challenge, we propose a modular, scenario-driven framework for generating synthetic datasets tailored to the evaluation of visual perception functions. The framework aligns with the operational boundaries and detection–response requirements of automated driving functions and comprises three stages: (1) configuring use-case-driven scenarios, (2) generating sensor data and ground truth via simulation, and (3) post-processing to ensure dataset usability. Designed to be generic and flexible, the framework is instantiated and demonstrated through its integration with specific platforms and tools, namely Pro-SiVIC and RTMaps. We evaluate the generated dataset from two perspectives, image fidelity and perception performance under synthetic weather conditions, in comparison to real-world conditions. Furthermore, we train multiple perception models under different learning paradigms, including baseline, transfer-learning, and mixed-training strategies, to examine the influence of synthetic data on robustness. Experimental results demonstrate not only the high quality of the generated data but also its effectiveness in evaluating visual perception functions, as well as its benefit to model robustness and generalization.

## 1. Introduction

With the advancement of autonomous driving technology, vehicle control is progressively shifting from human drivers to machine intelligence. Before SAE driving automation Level 3 (L3) [1] and higher systems can be deployed on public roads, their safety and reliability must be validated in complex traffic environments. One of the primary challenges arises from the virtually infinite range of driving situations encountered by autonomous vehicles in the real world, containing diverse environmental conditions, complex road scenarios, and dynamic interactions with various traffic participants, including rare and long-tail events. Robust evaluation, therefore, requires large-scale and representative test data. However, real-world driving alone is insufficient: as noted by Kalra and Paddock [2], demonstrating safety to a statistical degree would require billions of miles, posing enormous challenges in cost, time, and feasibility.

Perception plays a central role in autonomous driving, providing the environmental understanding required for downstream decision-making. Modern visual perception systems, mainly based on deep learning architectures, require large amounts of diverse, high-quality annotated data to perform accurately and robustly [3]. In practice, autonomous vehicles operate under constantly changing conditions, from daylight to nighttime, from favorable weather to heavy rain or fog, which introduce reduced visibility, noise, motion blur, and occlusions. Without sufficient exposure to such conditions during development and testing, perception models may suffer significant performance degradation when deployed in the field. This has motivated the development of several datasets that capture non-ideal conditions, such as Exclusively Dark (ExDark) dataset [4], Canadian Adverse Driving Conditions (CADC) dataset [5], and IDD-AW dataset [6], which have enabled researchers to train and evaluate visual perception systems under different adverse conditions. Despite their value, real-world datasets remain limited: data collection is constrained, rare events are scarce, and annotations in adverse conditions are difficult and error-prone. They also cannot easily provide the specific scenarios or annotation details required for different testing needs.

The above-mentioned limitations make synthetic datasets increasingly necessary as a complement to real-world datasets. Although synthetic datasets such as SYNTHIA [7], Virtual KITTI series [8,9], and SHIFT [10] have demonstrated the potential of simulation-based imagery for perception research, they still present several gaps. First, most existing synthetic datasets are generally organized around visual diversity and rendering realism rather than explicit operational requirements. As a result, the generated scenes are often weakly connected to Automated Driving System (ADS) use cases, Operational Design Domain (ODD) specifications, and perception evaluation objectives. Here, ODD defines the operating conditions under which an ADS is intended to function, while Object and Event Detection and Response (OEDR) describes the system capability to detect relevant objects and events and respond appropriately. Although some recent datasets, such as UrbanSyn [11], organize scenes according to ODD categories, this mainly remains a descriptive arrangement of environmental assets and conditions. Given the diversity of use cases, synthetic datasets often need to be customized for specific operational conditions. This requires a structured process that uses ODD specifications to guide scenario generation, yet current pipelines provide limited support for systematic and traceable coverage of these conditions. Second, existing synthetic dataset pipelines generally do not explicitly organize event conditions, behavioral triggers, and scene evolutions according to OEDR specifications. Consequently, complex driving interactions are often represented as relatively isolated scene configurations rather than temporally connected operational events. This limits the generation of datasets that can represent evolving traffic interactions and response-dependent driving situations, thereby reducing the ability to evaluate perception robustness under evolving driving conditions.

In the validation pipeline of ADS, real-world testing and virtual simulation play complementary roles. Large-scale projects increasingly adopt simulation-based evaluation, especially for adverse, rare, or safety-critical conditions that are difficult to reproduce physically. For example, the HEADSTART project [12] established a harmonized safety-validation methodology that integrates scenario-based simulation with real-world trials. The Hi-Drive [13] and AUGMENTED CCAM project [14] similarly employ virtual testing to explore challenging and boundary ODD conditions for high-automation functions, while SUNRISE [15] leverages simulation to assess system robustness and interoperability under degraded environments. These efforts clearly show that scenario-based simulation testing has become the standard and validated approach for ADS evaluation.

Recent studies have advanced scenario-based testing by developing ODD- and ontology-based approaches for generating logical and concrete test scenarios [16,17]. While these developments demonstrate the growing importance of ODD-driven scenario construction for system-level verification, they do not provide explicit mechanisms for structured synthetic dataset generation. In particular, current dataset generation workflows provide limited support for systematically translating use-case requirements and associated ODD/OEDR specifications into controllable synthetic datasets for data-driven evaluation and testing. In this context, we propose a scenario-driven synthetic dataset generation framework for visual perception evaluation. The framework guides the configuration of scenario attributes and structures based on the target use case and its associated operational conditions derived from ODD and OEDR specifications. It further enables controllable generation of synthetic data through the coordinated configuration of environmental and system factors, including adverse driving conditions. The main contributions of this paper can be summarized as follows:

1. A generic scenario-driven framework is proposed for synthetic dataset generation through configuration, generation, and post-processing stages under adverse driving conditions.
2. The framework provides a systematic and traceable configuration process linking use cases, ODD/OEDR conditions, testing scenarios, and evaluation objectives to controllable dataset generation.
3. The framework is instantiated using existing simulation platforms and toolchains, with representative examples provided for each stage.
4. The framework is applied to representative autonomous-driving use cases to generate the ADSceneSim dataset covering both urban and highway scenarios.
5. ADSceneSim is evaluated through comprehensive experiments, demonstrating its applicability for controlled robustness evaluation and as a supplementary training resource for visual perception models.

The remainder of this paper is structured as follows. Section 2 reviews related work on synthetic datasets and on scenario generation guided by ODD and OEDR. Section 3 presents the conceptual foundations of our scenario-driven framework. Section 4 details the design and implementation of the proposed framework. Section 5 applies the framework to real use cases and introduces the generated dataset. Section 6 reports the experimental evaluation. Section 7 concludes the paper and proposes future work.

## 2. Related Work

### 2.1. Synthetic Datasets for Autonomous Driving

A large number of synthetic datasets have been introduced over the past decade to alleviate the cost, safety constraints, and coverage limitations of real-world data collection in autonomous driving. Some of the earliest efforts were dedicated to controlled adverse-weather scenarios, such as FRIDA [18], generated using a private simulator to provide scenes rendered under different types of fog. As simulation platforms developed, researchers began constructing more complex urban environments for semantic perception tasks. Datasets such as SYNTHIA [7] and VEIS [19], produced using the Unity engine, demonstrated that virtual worlds can generate large-scale pixel-perfect labels for tasks such as semantic segmentation and object detection. Similarly, GTA-V datasets [20] showcased rapid scene generation and diverse city structures, though they remain fundamentally constrained by the rendering capabilities and asset libraries of commercial game engines, leading to limited photorealism, repeated scene structures, and incomplete support for adverse weather.

Procedural-rendering and simulator-driven datasets later emerged, with CARLA becoming a key platform enabling full control over sensor suites, weather, illumination, and traffic dynamics. This led to the development of datasets such as IDDA [21], CarlaScenes [22] and the more recent AmodalSynthDrive dataset [23], which broaden task coverage to depth estimation, multi-view fusion, and domain-shift analysis. In parallel, the Virtual KITTI series [8,9] introduced photorealistic, cloned 3D reconstructions of real KITTI scenes [24], providing multi-annotated stereoscopic RGB sequences with depth, optical flow, and tracking labels. These synthetic yet visually realistic sequences were specifically designed to support multitask perception and domain-shift benchmarking. More recent efforts, such as SHIFT [10] and Adver-City [25], further expanded environmental diversity by modeling continuous domain variations, particularly under different weather conditions, for targeted perception evaluation.

High-fidelity synthetic datasets, such as Synscapes [26], UrbanSyn [11], and RealDriveSim [27], have advanced visual realism by relying on curated asset libraries and offline path-traced rendering that more closely approximate real-world appearance. In parallel, these datasets broaden task coverage by providing richer multi-modal annotations, including LiDAR, scene flow, and fine-grained semantic labels. However, despite these improvements in realism and annotation diversity, most synthetic datasets remain fundamentally content-driven: they focus on generating visually diverse scenes but usually do not provide well-defined scenario structures, ODD-aligned condition definitions, or controlled variations of safety-critical environmental and behavioral factors. As a result, they offer limited support for evaluation settings that require systematic scenario coverage rather than unconstrained visual diversity.

Recent advances in learned generative models have introduced new paradigms for driving-scene synthesis. Approaches such as DriveDreamer [28] employ diffusion-based generative modeling to generate realistic driving environments and controllable environmental variations from real-world driving data. World-model-based approaches, including Vista [29], Drive-WM [30], and GAIA-1 [31], further model temporal traffic evolution and interactive driving dynamics for long-horizon simulation and prediction. These approaches significantly improve visual realism, scene diversity, and controllable environment synthesis, reducing dependence on manually constructed simulation assets and handcrafted rendering pipelines. Many learned generative models further support controllable variations in weather conditions, scene layouts, traffic participants, and temporal dynamics, enabling scalable generation of diverse driving environments and interactions. Nevertheless, these approaches are mainly focused on realistic scene synthesis and dynamic simulation, while still providing relatively limited support for structured scenario specification and dataset generation driven by operational conditions and use case requirements. As a result, the integration of learned generative approaches into systematic and traceable workflows for dataset generation and perception evaluation under adverse driving conditions remains underexplored.

### 2.2. Scenario Generation with ODD/OEDR

Scenario-based simulation has become a widely adopted methodology for evaluating ADS. Ref. [32] established formal definitions of scene, situation, and scenario, providing a semantic basis for modeling dynamic traffic environments. Subsequent research [33,34] introduced structured scenario taxonomies and the Functional–Logical–Concrete (F–L–C) decomposition, allowing testing workflows to derive parameterized logical scenarios from abstract functional descriptions. To support the practical representation of such scenarios in simulation, several standards have been developed. OpenDRIVE [35] defines road geometry, lane topology, and infrastructure elements; OpenSCENARIO [36] specifies actor behaviors, events, and triggers; and OpenLABEL [37] provides a unified format for annotation and labeling. These standards are widely implemented in simulators such as CARLA [38] and SVL Simulator [39], enabling reproducible and cross-platform scenario execution.

Although these simulators provide configurable scenarios, sensor simulation, and automated annotation capabilities, they are primarily designed for scenario execution and data generation rather than the systematic organization of synthetic datasets. In existing workflows, scenario configurations for dataset generation are often defined separately from use case requirements and operational specifications. Consequently, existing workflows provide limited support for maintaining traceable relationships between operational conditions and generated datasets, systematically covering driving conditions, and organizing dataset composition according to evaluation objectives.

To improve the consistency between operational requirements and scenario configuration, ODD is increasingly used to constrain scenario creation by specifying the environmental and traffic conditions under which automated functions operate. Scenario-based systems engineering positions ODD as a structural element for defining system capabilities and selecting relevant scenarios across abstraction layers [40]. ODD also provides the boundary conditions for identifying and classifying critical scenarios, guiding how triggering events and safety-relevant situations are derived in systematic mapping studies [41]. Recent work further integrates ODD elements with a system’s behavioral competencies, applying filtering and rule-based construction to generate logical scenarios that reflect the system’s intended operating domain [42]. At the simulation level, logical scenario generation further uses ODD to define maneuver types, parameter ranges, and hazard boundaries following ISO standards [43]. In addition to research efforts, standardization initiatives such as OpenODD [44] formalize ODD descriptions to support consistent scenario definition and exchange across tools and organizations. These works provide a strong foundation for ODD-guided scenario-based testing; however, most remain primarily focused on system-level validation, serving verification and safety-assessment processes rather than data-oriented evaluation needs.

The OEDR concept defines the objects, events, and corresponding system reactions that an automated driving system must handle. In the National Highway Traffic Safety Administration (NHTSA) report on ADS scenarios and testable cases [45], OEDR capabilities are explicitly identified as one of the four core elements that define a test scenario. Recent reviews [46,47] recognize OEDR considerations as an essential part of the functional context for scenario definition. However, existing implementations generally use OEDR as a conceptual reference for scenario definition and system-level testing, whereas its operational events and behavioral interactions are rarely organized as structured components for dataset generation.

## 3. Concept Foundations

Synthetic datasets for evaluating visual perception systems, especially in safety-critical domains such as autonomous driving, need to go beyond replicating real-world appearance. They require a principled foundation that supports the generation of structured, realistic, and task-relevant data, enabling comprehensive testing across diverse operational conditions. This section introduces the conceptual dimensions that form the basis of our scenario-driven synthetic dataset framework. Section 3.1 explains how operational and behavioral constraints define the representational space in which valid scenarios can be modeled. Section 3.2 describes the semantic structure used to compose scenes and organize their spatial and temporal elements. Section 3.3 establishes the principles that enable scenarios to be systematically controlled and varied for targeted evaluation. Section 3.4 discusses how consistent and geometry-aligned labels are generated to support multiple perception tasks. Together, these dimensions provide the theoretical foundation for the modular implementation architecture presented in Section 4.

### 3.1. Scenario Modeling Based on ODD and OEDR

A comprehensive synthetic dataset should reflect the range of operational and behavioral conditions under which an autonomous system is expected to function [48]. In practice, these conditions are commonly described through use cases and their associated ODD specifications, which define the operational conditions under which an ADS is intended to perform the Dynamic Driving Task (DDT) [1]. Complementarily, OEDR specifies the perception and response capabilities required from the system, including object/event detection and associated reactions.

Building on these definitions, ODD and OEDR specify the environmental conditions and system-level requirements that should be incorporated into scenario construction. These conditions and constraints form the foundational basis for dataset generation, where scenarios are configured by integrating this information. Scenario construction draws on environmental factors and their associated parameters, such as road layout and weather, to ensure that each scene is realistic and coherent. In parallel, system-level parameters, such as sensor placement and actor roles, are also included and instantiated to simulate the perception system and the corresponding response mechanisms or modules. At the same time, scenario configuration also embeds OEDR-defined interactions as system elements by defining controlled events and triggers. Scenario configuration connects the structural diversity defined by ODD with the behavioral complexity outlined in OEDR. Each generated scene reflects a valid operational context while explicitly targeting perception-related responsibilities.

### 3.2. Semantic Structure for Scenario Composition

In this subsection, we introduce a layered semantic model for scene composition as one of the conceptual foundations of our framework, which is inspired by [32] and also applied in French PRISSMA project [49]. Each scenario is constructed as a sequence of temporally evolving scenes, where each scene is defined by a semantically coherent and hierarchically structured layout. This layout reflects not only the physical configuration of the environment but also the roles and interactions of dynamic agents within that context.

The semantic structure for scenario composition consists of four interdependent components. The first component, scenery, refers to the static spatial background of the scene. It includes the lane network, defining the topology of road segments, intersections, and drivable areas, as well as static elements such as traffic signs and road markings. It also incorporates global environmental conditions such as weather and lighting. The second component, dynamic elements, represents mobile agents such as vehicles and pedestrians. Each agent is assigned a semantic role and corresponding behavioral logic (e.g., following, merging, crossing), enabling scenes to reflect plausible traffic interactions. The third component, actors and observers, extends both dynamic and static elements by assigning perception roles. Unlike the real world, where perception is limited to individual viewpoints, simulation allows comprehensive and uncertainty-free observation from a global perspective. The final component, scene-level logic, governs the temporal evolution of a scenario through the definition of events, actions, and criteria. An event serves as a trigger (e.g., proximity, timing, environmental change) that initiates an action by a specific agent. The action defines the agent’s response, while the criteria specify the conditions for scenario completion, such as reaching a goal, meeting safety constraints, or satisfying time limits. This logic component ensures control over the progression of the scenario, and enables the reuse of scenario templates across different configurations. This model acts as an intermediate representation between the semantic scenario structure and downstream configuration files.

### 3.3. Design Principles for Scenario Controllability

Scenario controllability refers to the ability to manage scene elements and interactions so that perception models can be tested under systematically varied conditions. This capability is essential for stress testing and reproducible evaluation of perception systems. To support this requirement, the proposed framework is built on three key conceptual principles: configuration decoupling, parameterization, and event-driven execution.

First, configuration decoupling separates environment settings and system properties into independent layers (as in Section 3.1). This modular structure allows users to adjust environmental variables (e.g., road layout, lighting, weather), sensor configurations (e.g., sensor placement and capabilities), and event dynamics (e.g., conditions, logic, and actions) in isolation or combination, enabling high-dimensional control over the scene. Second, parameterization defines which variables can be controlled and sets their valid ranges. This enables systematic variation of specific factors while keeping others fixed. For example, weather can be parameterized by type (clear, rain, fog), intensity (light, medium, heavy), and time of day (day, dusk, night), allowing targeted evaluation of perception robustness. Third, event-driven execution controls how scenes evolve. As discussed in Section 3.2, events are defined within the scene-logic component and must be linked to specific trigger conditions to be activated. These triggers are typically based on spatial, temporal, or contextual conditions, and activate actions such as lane changes, occlusions, or sudden weather transitions. This mechanism allows dynamic variations to unfold in a controlled and repeatable way.

### 3.4. Consistent Multi-Task Label Generation

The consistency of cross-task annotation is grounded in a unified, geometry-aware representation that supports annotation across multiple perception tasks. The simulation environment provides rich and structured references, which can include object-level attributes (such as position, classification, and dimensions), color-rendered semantic maps, depth maps, and information about scene-level elements (such as road topology, traffic infrastructure, and environmental conditions). All references are aligned into a shared spatial coordinate system (e.g., projected by using known sensor parameters within the simulator), which allows different annotations to constrain and reinforce one another. For example, object-level geometry guides instance mask construction, depth information supports visibility filtering, and scene layout constrains semantic labels such as drivable areas. These interactions contribute to richer annotations and also ensure that different task-specific labels accurately follow the scene’s geometric layout and semantic content.

## 4. Framework Design and Implementation

In this section, we present the design and implementation of our scenario-driven synthetic data generation framework. Building upon the conceptual foundations outlined in Section 3, the framework is implemented through a modular architecture composed of three core stages. Section 4.1 presents the scenario configuration based on ODD and OEDR, where high-level descriptions are structured into simulation-ready configurations. Section 4.2 covers the automatic execution and data generation, which deploys these configurations within the simulator and associated tools to capture the raw simulation outputs. Section 4.3 describes the post-processing stage for annotation across multiple perception tasks. Each stage is implemented through dedicated configuration or execution methods, supported by platforms and tools that manage simulation, control, and annotation workflows. The objective is to demonstrate the practical feasibility of the proposed framework and to provide a transferable reference for deploying it with other platforms and toolchains.

### 4.1. Scenario Configuration

**Figure 1:**
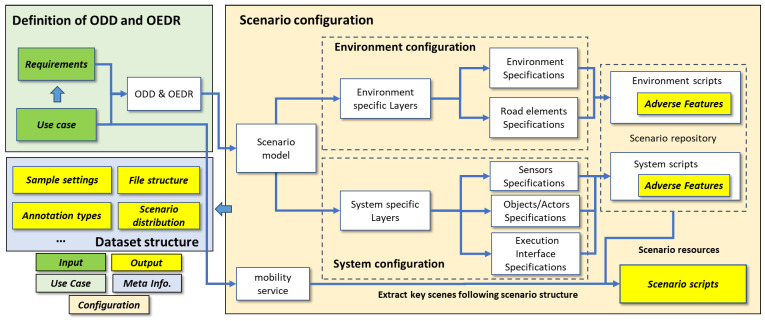
Structural design of the configuration phase in the proposed framework.

As shown in Figure 1, the architecture serves as the configuration backbone of the proposed framework by transforming high-level concepts and requirements into structured, simulation-ready components. This transformation begins with the specific use cases and relevant requirements, which specify key aspects including expected operational behaviors, required perception tasks, and the environmental variability the autonomous system is expected to handle. Based on these inputs, an appropriate taxonomy, such as those proposed in [50,51,52], is then chosen and adjusted to structure the ODD and OEDR models. As discussed in Section 3, the ODD provides a structured representation of the physical, environmental, and traffic contexts in which the system is expected to operate safely, while the OEDR defines the key objects, events, detection tasks, and system responses involved in perception and control workflows. Together, they serve as foundational references for the configuration phase.

**Figure 2:**
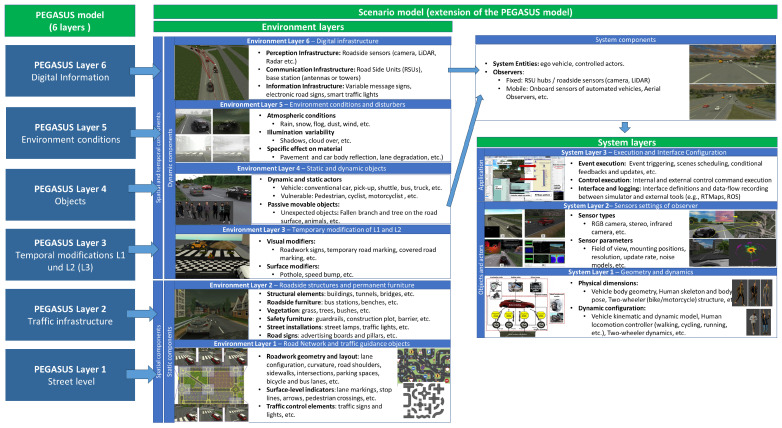
Scenario model adapted from PEGASUS with system-level extensions.

Based on the ODD and OEDR specifications, the configuration process constructs a scenario model that decomposes scenario content described within the operating domain. This model is inspired by the paradigm introduced in the PEGASUS project [34], which proposes a layered representation of scenarios to ensure systematic coverage across testing dimensions. In our framework, this model is extended to support simulation and is structured, as shown in Figure 2, into two primary branches: the environment layers and the system layers.

As illustrated in the left portion of Figure 2, the environment layers within the configuration architecture are directly derived from the PEGASUS scenario model with the six layers originally proposed. In the original taxonomy, these layers correspond respectively to the road network (Layer 1), roadside infrastructure (Layer 2), temporary traffic modifications (Layer 3), movable objects (Layer 4), environmental conditions (Layer 5), and digital information (Layer 6). Based on this foundation, the environment layers in our framework describe the external configuration of the driving scene by integrating both static and parameterized dynamic components, including road geometry, infrastructure elements, temporary modifications (e.g., lane closures or construction zones), objects distributed throughout the scene, and environmental variations such as fog, rain, or illumination changes. The environment layers also incorporate digital infrastructure, such as communication units and roadside sensors, which form the static digital layer of the scene. This layered structure allows flexible control of spatial and environmental factors to generate scenes under both nominal and adverse conditions for evaluating perception robustness.

While the PEGASUS methodology was originally developed as a systematic framework for scenario-based testing of automated driving functions, its multi-layer model also provides a structured taxonomy for organizing environmental and spatial conditions, which forms the basis of the environment layers described earlier. However, it does not explicitly address the internal configurations of the objects or the sensor-equipped actors that are essential for simulation-based perception evaluation. To bridge this gap, our framework introduces an extended branch of system layers, reorganizing elements from PEGASUS Layers 4 and 6 into three system layers. The lowest system layer (Layer 1) captures the physical characteristics of the ego vehicle and other actors. The intermediate layer (Layer 2) defines the perception configurations, including sensor types and parameters that determine observability under varying conditions. The highest layer (Layer 3) acts as an operational bridge between the internal simulation environment and externally defined control logic. It provides structured interfaces for scenario scheduling, control feedback, and data exchange with other platforms (e.g., RTMaps^TM^, ROS). Through this layer, high-level execution policies can be applied to control agent behavior and drive scene evolution.

The full scenario configuration emerges from the integration of these two structural branches (as in Figure 1). Environment and system layers are first translated into detailed configuration specifications, which are then written into executable scripts. These scripts serve as low-level directives that instantiate and assemble the scenario layers within the simulation engine. Notably, both types of scripts support the integration of adverse conditions, such as adverse weather conditions, sensor noise, or conflicting traffic participants, that are vital for evaluating perception robustness. In our implementation, these scripts are defined using a proprietary syntax and parsed through dedicated C++ interfaces within Pro-SiVIC^TM^. While this scripting format is simulator-specific, the underlying configuration structure, organized through layered design, remains transferable. It can be extended to support open standards such as OpenDRIVE for environment modeling and OpenSCENARIO for actor behavior and interaction logic, enabling broader compatibility with standardized simulation toolchains.

After the layered configuration (both environment and system) has been translated into executable scripts, they are collected into a scenario repository that provides the reusable resources for subsequent instantiation of simulation scenarios. As outlined previously, the use cases define the high-level operational objectives and contextual constraints of the system under test. From the use case, functional services are derived as in Figure 1, describing the specific system behaviors required to fulfill these objectives (e.g., safe passenger pick-up, lane-merging assistance). These functional services are then mapped to key scenes that correspond to the semantic scenario structures defined in Section 3.2 and capture the essential scenes and interactions to be reproduced in simulation. The key scenes guide the selection of environment and system scripts from the scenario repository, thereby forming scenario-level descriptions, i.e., structured scenario scripts. Within this structure, the main scenario script acts as the central control unit, integrating the environment and system scripts and managing their execution during simulation runtime.

Finally, beyond their role in simulation execution, these scenario scripts also provide the foundation for constructing the dataset. At the operational level, the scenario scripts determine how simulated data streams are sampled and recorded during simulation runs. The broader design of the dataset, however, is also shaped by requirements derived from the ODD coverage criteria and the OEDR-based perception and response specifications that guide evaluation. Accordingly, the dataset construction process can be structured along four dimensions. The first dimension, the ODD-based condition distribution, specifies the relative proportions of environmental conditions, traffic situations, and object categories to be represented in the dataset. The second dimension is defined by the settings specified within the system scripts, which determine recording modalities, sampling rates, and capture durations, ensuring the consistent generation of time-synchronized multimodal data streams. The third dimension, the annotation requirements derived from the OEDR model, defines the types of labels needed for evaluation, including object detection, segmentation, tracking, and event-level annotations within a unified ontology. The fourth dimension concerns the data organization stage and structures the resulting data streams, annotations, and metadata into a well-defined file structure that supports efficient storage, retrieval, and interoperability across perception tasks, toolchains, and evaluation pipelines.

### 4.2. Simulation Execution and Data Generation

The execution and generation layer of the proposed framework converts the executable configuration files into simulation runs. It then captures simulated sensor data together with reference information, which form the basis for generating multiple types of annotations during subsequent post-processing. The layer consists of five functional components: (1) environment and system modelling, (2) adverse condition modelling, (3) simulation deployment, (4) interactive event control, and (5) sensor data and reference capture. In combination, these components support the construction, execution, and data collection of scenario- and system-specific simulations. This layer is designed to be conceptually platform-agnostic, aiming to support integration with diverse simulation engines and toolchains. In our implementation and use case studies, we employ Pro-SiVIC^TM^ as the high-fidelity simulation engine and RTMaps^TM^ for runtime control, data logging and replay, and the integration of autonomous driving functions.

#### 4.2.1. Environment and System Modelling

**Figure 3:**
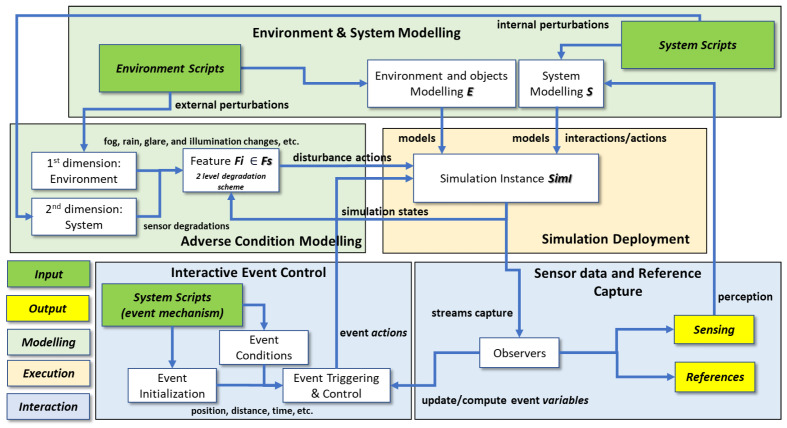
Structural design of the generation phase in the proposed framework.

As illustrated in Figure 3, the execution process begins with the environment and system modelling module, which instantiates the layered configurations introduced in Section 4.1. At the configuration stage, the parameterized environment and system layers have already been translated into executable scripts. The scenario and system modelling module parses these scripts and instantiates them into concrete simulation entities *E* and *S*, and provides the executable foundation upon which subsequent modules operate.

On the environment side, road layouts, roadside infrastructure, and traffic flow patterns are built from parameterized configuration files that describe spatial structure and dynamic conditions. The goal is to reproduce the complexity and diversity of real-world driving situations in a controlled and repeatable manner. Pro-SiVIC^TM^ is employed to construct and render realistic and detailed virtual environments, containing road networks, traffic conditions, weather phenomena, pedestrians, vehicles, and roadside objects. In our implementation, the environment library primarily consists of 3 simulated environments representing test track, highway, and urban traffic conditions. Within this library, the test track environment corresponds to the digital twin of the Satory test track.

On the system side, the ego vehicle and surrounding actors are modelled with both their physical representations and behavioral dynamics. Within the implemented framework, the ego vehicle is equipped with a sensor suite including cameras, LiDAR, Radar, and GNSS/IMU, whose placement and parameters replicate the realistic configuration of the robotic Renault Zoe used in the PRISSMA and AUGUMENTED CCAM project. To ensure physical fidelity, Pro-SiVIC^TM^ integrates a comprehensive vehicle dynamics model covering the car body, suspension, wheels and tires, powertrain, and steering system. Interactions with the ground and other objects are computed through the simulator’s embedded ray-tracing engine. For the control of simulated actors, various modes are available, including manual driving, trajectory-following control, direct command-based control, and closed-loop control via RTMaps^TM^, where high-level decision and control modules are deployed.

#### 4.2.2. Adverse Condition Modelling

The adverse condition modelling module implements a two-dimensional degradation scheme: one that alters the external environment and another that affects the system (sensor model) itself, as shown in the corresponding branch of Figure 3. In our implementation, this module primarily focuses on weather-induced degradations effects, since adverse weather is one of the most influential factors affecting both visibility and sensor performance in autonomous driving. At the environmental level, the framework is designed to model various physical conditions that reduce visibility, such as fog, rain, glare, and illumination changes. At the system level, it is designed to represent how sensors are affected under these adverse conditions, for example, by introducing noise, limited dynamic range, or optical distortions. The combination of the two levels of perturbation ensures that visibility and sensor fidelity are both challenged during simulation, and can be formalized as a perturbation feature set $F_{s}=\left\{F_{i}|i=1,\ldots ,n\right\}$ containing all degradation factors injected into the simulation instance.

**Figure 4:**
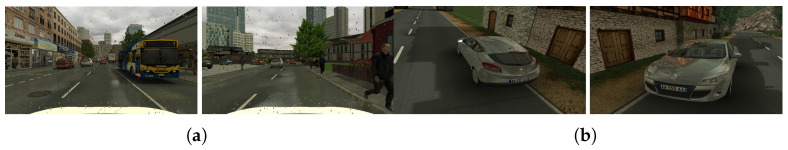
Simulated reflection effects on pavement and carbody surfaces. (**a**) Pavement (planar reflection rendering); (**b**) Carbody (cube map reflection rendering).

**Figure 5:**
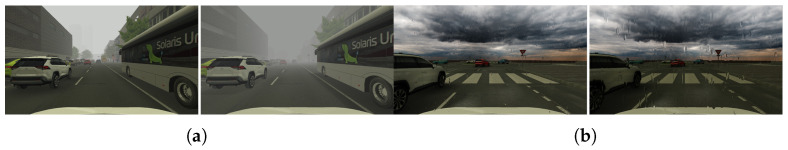
Visual effects under adverse weather: fog and rain scenarios with increasing severity. (**a**) Fog conditions: light and dense intensities; (**b**) Rain conditions: droplets in air & on windshield.

In our implementation and use case studies, the above-mentioned factors are realized in Pro-SiVIC^TM^. On the environmental side, fog is implemented using depth-dependent attenuation derived from Koschmieder’s Law. Reflection effects are also supported to enhance visual realism under adverse conditions. These effects are generated using planar and cube-map reflection techniques, with Figure 4 showing two examples: pavement reflections and carbody reflections. When simulating rain, these reflection mechanisms are activated and combined with particle-based rain modelling. Examples of fog and rain at different severity levels are shown in Figure 5. Glare effects are represented by introducing overexposed regions, whereas global tone transformations emulate variations in ambient illumination, such as overcast skies or shaded environments. These perturbations are rendered through a multi-filter mechanism [53], which allows multiple disturbances to be composed in parallel.

On the system side, the employed simulation engine provides a physics-inspired rendering pipeline for camera modelling that enables both optical and sensor-level perturbations. Sensor noise is introduced to simulate electronic interference under low-light conditions, while HDR rendering, combined with automatic exposure control, reflects the limited dynamic range in strong illumination contrasts. Depth of Field (DoF) effects and motion blur further simulate optical limitations, and raindrop occlusions on the windshield reduce image quality through local distortions and transparency loss.

#### 4.2.3. Simulation Deployment

The simulation deployment module (light yellow rectangle in Figure 3) serves as the execution backbone of the framework, bridging the transition from static environment and system modelling to a running simulation instance $SimI(E,S,F_{s})$. In practice, its functionality can be structured into three core responsibilities. First, once the static modelling is complete, the module manages the scheduling of simulation runs, which includes initializing the ego vehicle’s dynamic state, generating background traffic flows, and assigning environmental parameters. Second, it establishes temporal synchronization across heterogeneous components, ensuring that vehicle dynamics, perception sensors, and higher-level functional modules evolve under a common simulation clock. Third, it integrates with external middleware to support sensor data streaming, the logging of reference information, and online interaction with autonomous driving functions.

In the implementation and use case studies, simulation deployment is realized through the combined use of Pro-SiVIC^TM^ and RTMaps^TM^. Within Pro-SiVIC^TM^, the static scenario models established during the modelling stage are promoted into runtime execution, where the engine activates the preconfigured system and environment conditions and executes high-fidelity vehicle dynamics and environment rendering. RTMaps^TM^ complements the deployment process by providing the runtime environment for higher-level functional modules and managing their bidirectional communication with Pro-SiVIC^TM^ via simulation I/O interfaces. It also ensures temporal synchronization across vehicle dynamics, sensor outputs, and higher-level functional modules. In addition, RTMaps^TM^ supports systematic data logging and replay, thereby ensuring reproducibility for closed-loop experimentation.

#### 4.2.4. Interactive Event Control

The interactive event control module extends the runtime instance with an event-driven mechanism that introduces real-world uncertainties and dynamic interactions into the simulation. As illustrated in the left light blue rectangle of Figure 3, this mechanism is specified within the system scripts (introduced in Section 4.1), which describe key elements such as event initialization, conditions, and triggering logic. These scripts are then loaded into Pro-SiVIC^TM^ for execution.

During execution, output streams from the simulation engine are forwarded to RTMaps^TM^ as observation or sensor data. The scenario manager, integrated as a module within RTMaps^TM^, continuously computes and updates event variables, ranging from basic kinematic quantities (e.g., position, velocity) to higher-order variables, including derived risk measures and contextual conditions. When the predefined conditions are satisfied, the corresponding events are triggered, leading to scene modifications such as docking maneuvers at bus stations, the sudden appearance of obstacles, or emergency braking. The event commands are transmitted back to Pro-SiVIC^TM^ via middleware interfaces (e.g., Data Distribution Service (DDS) or First In First Out (FIFO)) and executed in real time, thereby enabling modifications to both dynamic and static aspects of the scene. As a result, this mechanism allows the simulation to reproduce time-sensitive and safety-critical interactions, which are essential for evaluating perception robustness.

#### 4.2.5. Sensor Data and Reference Capture

Finally, the sensor data and reference capture module is tasked with acquiring multimodal sensor outputs and aligning them with structured reference information, as shown in the right light blue rectangle of Figure 3. The sensor data streams, which include simulated RGB imagery and preliminary semantic renderings (produced by assigning predefined colors to material classes), are captured in real time and transferred into RTMaps^TM^ for subsequent post-processing. In parallel, reference attributes, such as object identifiers, spatial positions, semantic classes, and motion states, are recorded. These outputs are used for annotation postprocessing and subsequently for evaluating visual perception models across multiple tasks, including object detection, semantic and instance segmentation, and multi-object tracking.

### 4.3. Annotation Post-Processing

**Figure 6:**
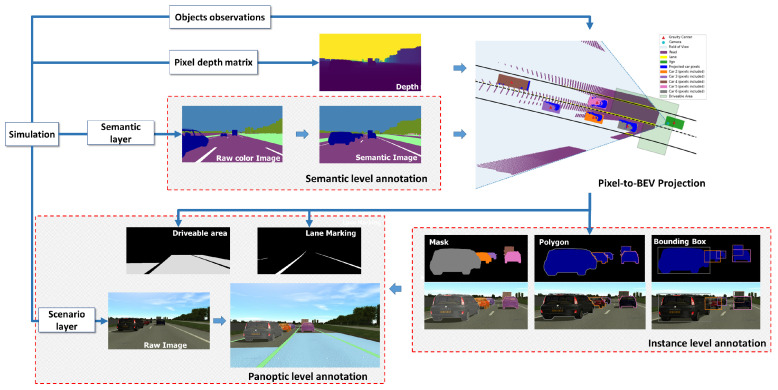
Process of multi-level ground truth generation from simulation, including semantic, instance, and panoptic annotations.

**Figure 7:**
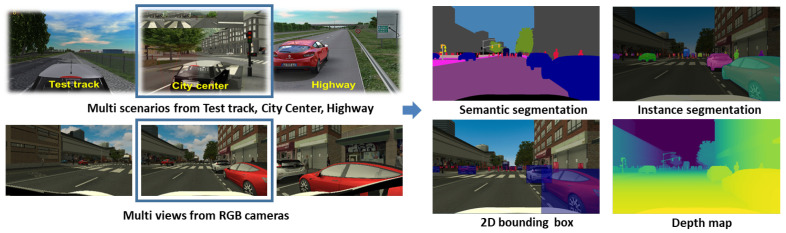
Scenario capture and multiple annotations generated by the implemented framework.

High-quality and diverse ground-truth annotations are essential for training and evaluating perception models in autonomous driving. Different from manually annotated real-world datasets, the simulator automatically generates rich and precise scene-level reference signals, including semantic masks, depth maps, object metadata, etc. In simulation, these references are internally consistent and strongly correlated. By using this property, we introduce a hierarchical post-processing framework (Figure 6) that converts simulation references into semantic-, instance-, and task-level annotations. The right panel of Figure 7 illustrates representative examples produced by the framework.

#### 4.3.1. Multi-Level Annotation

Semantic annotations are obtained by decoding the preliminary semantic renderings (as described in Section 4.2.5) using a predefined color-to-index table, filtering invalid pixels, and remapping them to the label scheme used by public benchmarks. The resulting semantic masks also support tasks such as instance segmentation and drivable-area extraction.

Instance annotations are produced through a projection module that combines semantic segmentation maps with object-level metadata in a unified Bird’s Eye View (BEV) representation. Semantic pixels are paired with depth values and back-projected into BEV space using camera intrinsics and extrinsics. In parallel, object footprints are reconstructed in BEV space from metadata (position, dimensions, orientation), and projected pixels are associated with their corresponding footprints. Because instance regions (pixels) are determined by geometric footprints in BEV space rather than depending on the object regions in the semantic mask, where adjacent objects belonging to the same category may appear merged, the framework produces accurate object masks and bounding boxes even when objects are crowded or occluded. Track identifiers and motion trajectories are also preserved across consecutive frames, which is essential for sequential tasks such as multi-object tracking, behavior prediction, and risk assessment.

Task-oriented annotations are generated by integrating semantic cues, instance regions, and prior knowledge of road topology. Lane structures and drivable surfaces are inferred by combining static structural elements (e.g., lane boundaries, road geometry) with the spatial occupancy of dynamic agents (e.g., vehicles, pedestrians) in BEV space, producing driving-relevant supervision suitable for downstream perception modules.

#### 4.3.2. Visibility-Aware Annotation Strategy

Adverse environmental conditions may render parts of the scene almost invisible, raising the question of whether annotations should include such objects. In real-world datasets, such instances would not be labeled, but simulation retains them because scene geometry is fully known. When such imperceptible instances remain in the annotation, a mismatch emerges between the image evidence and the ground truth. For the training phase, the model receives supervision for objects without usable visual cues, introducing noisy signals that degrade representation learning [54]. During evaluation, the model is penalized for missing targets outside the perceivable range, producing an unfair performance estimate. To address this, we adopt a visibility-aware annotation strategy that aims to ensure that labels correspond only to physically perceivable targets, especially in adverse weather conditions.

For foggy scenes, since our fog rendering inherently follows Koschmieder’s Law, the fog density parameter allows us to derive the maximum distance at which objects remain perceptually visible. At greater distances, object contrast falls below a perceptual threshold, and targets can no longer be separated from atmospheric light. By using this visibility limit, we remove the annotations of instances that lie outside the fog-limited perceptual range, ensuring that annotations remain aligned with the actual visual constraints imposed by the simulated fog.

For rainy scenes, we consider that visibility is degraded mainly by localized texture loss caused by raindrop streaks, motion blur, and surface reflections. Instead of using a physical distance threshold, we assess visibility directly at the image level by comparing rainy patches with their clear-weather reference captured under the same scene configuration. Structural similarity (SSIM map in [55]), edge response (Canny edge density in [56]), and gradient magnitude (Sobel gradient in [57]) are jointly examined to detect objects whose contours and textures are largely washed out under rain conditions. Objects that fail these appearance-based visibility checks are removed or marked as ignored, preventing the training and evaluation phases from being influenced by these targets.

### 4.4. Framework Generality and Transferability

Although the current implementation is developed using Pro-SiVIC^TM^ and RTMaps^TM^, the proposed framework is not restricted to a specific simulator or software ecosystem. The ODD/OEDR-driven scenario configuration process, layered environment and system modeling, event-driven execution logic, dataset generation pipeline, and annotation post-processing stages are designed independently from the underlying rendering and execution mechanisms. In the current implementation, Pro-SiVIC^TM^ is mainly used for environmental rendering, sensor simulation, and vehicle dynamics integration, while RTMaps^TM^ handles runtime execution, data recording, and integration of external autonomous-driving modules. Distributed data exchange and synchronized sensor streaming are achieved through FIFO and DDS communication interfaces, enabling bidirectional interaction between external modules and the simulation environment during runtime. Consequently, transferring the methodology to alternative simulation ecosystems mainly requires adapting the execution, synchronization, communication, and external-module integration layers while preserving the same configuration process and dataset generation workflow.

More specifically, the proposed architecture separates scenario configuration, simulation execution, and inter-module communication into distinct functional layers. The environment layer describing road geometry, lane topology, infrastructure elements, and static scene components can be mapped to OpenDRIVE representations or equivalent road-network formats. The system layer governing actor behaviors, interactions, triggering conditions, and event sequencing can be represented using OpenSCENARIO descriptions and executed in platforms such as CARLA, CARMaker [58], and LGSVL.

RTMaps^TM^ is currently used for runtime execution and external-module integration. Similar functionalities may also be implemented using ROS/ROS2, Apollo Cyber RT, Autoware, or equivalent distributed execution frameworks supporting modular autonomous-driving workflows through standardized communication interfaces. The FIFO and DDS communication infrastructure can also be replaced by alternative middleware solutions such as FastDDS or ZeroMQ, enabling synchronized data exchange between heterogeneous modules across different processes, machines, or simulation environments throughout the generation pipeline.

The handling of adverse conditions follows the same layered organization principle. Adverse situations are introduced during the scenario configuration process through operational descriptions, such as weather conditions and sensor perturbation constraints, while their physical realization is handled by the underlying simulation engine. Although rendering models and implementation details may differ across simulation platforms, the proposed organization process for configuring and integrating adverse conditions remains unchanged. Furthermore, the annotation post-processing stage remains decoupled from the simulator implementation. Although annotation mechanisms may vary across platforms, the proposed approaches, including multi-level annotation and visibility-aware processing, can still be applied by adapting the existing annotation and metadata.

## 5. Case Study on Framework Application

### 5.1. Use Case Context

In the case study section, the implemented framework is demonstrated across multiple autonomous driving use cases drawn from French PRISSMA and European AUGMENTED CCAM projects. The Bus Station Automated Service (BuSAS) represents a PRISSMA urban use case focused on ego-vehicle docking, obstacle handling, and degraded-condition management around predefined bus stations. In addition, two highway use cases from the AUGMENTED CCAM project are included to represent highway scenarios involving merging operations and temporary road-work situations. All systems are initially tested at the Satory test track in Versailles, coupled with a digital twin for cross-comparison between physical and virtual experiments. While this hybrid setup supports complete system validation, the limited diversity of the physical sites and their digital twins constrains the evaluation of perception robustness across diverse visual and environmental conditions.

Both urban and highway use cases considered in this study have ODD that extend beyond what the controlled test track can represent. BuSAS ODD includes urban operating environments, aligning in scope with the Paris2Connect PoC (Proof of Concept) in PRISSMA, which explores similar automated mobility services in open urban corridors. Likewise, the ODDs of the AUGMENTED CCAM use cases include highway-level situations that also cannot be fully replicated on a single physical site. As a result, although the generated synthetic dataset exceeds the representation of the test track site and corresponding digital twin, its design is constrained by the system’s defined ODD and OEDR boundaries, ensuring that all simulated scenarios stay functionally relevant and aligned with real-world operation and safety requirements.

### 5.2. Scenario Configuration for the Case Study

The configuration framework introduced in Section 4.1 is instantiated for the adopted use cases to generate structured scenarios. In this study, the ODD follows the specifications defined in the PRISSMA final POC deliverable [59] and the AUGMENTED CCAM deliverable [60], where it was derived by applying the generic taxonomies proposed in [51,52] to the use cases and their operational requirements. These taxonomies translate these high-level descriptions into a formal ODD specification by classifying relevant operational factors into 6 main categories: physical infrastructure, scenery, environmental conditions, traffic conditions, digital infrastructure, and vehicle capabilities. However, this taxonomy was developed primarily for system validation. For this reason, the present work adapts and extends it for dataset generation. To support this extension, additional details are added, mainly in the environmental dimension, to describe different levels of weather severity and related effects, including weather-induced conditions (e.g., reflections on the roadway or vehicle body, surface wetness) and illumination-induced conditions (e.g., glare, shadow).

In the corresponding deliverables, OEDRs detail the responses of the system for detected situations, organizing them into four main categories: (1) Obstacle-related events, covering interactions with lead, adjacent, and opposite vehicles, as well as vulnerable road users such as pedestrians; (2) Ego events, including operating outside its defined ODD or failure modes requiring driver intervention; (3) Road-related events, such as surface condition and marking visibility; and (4) Environmental events, including variations in weather, illumination, and traffic signals. Each event is associated with a required system response and safety constraint, for instance, adapting speed and inter-vehicle distance under fog or rain conditions, or stopping when a pedestrian is detected within a Time to collision (TTC) threshold of 1 s. These OEDR definitions provide the perceptual and behavioral logic underpinning scenario configuration and dataset generation.

The implemented configuration of scenario models for all use cases is summarized in Table A1 and Table A2 in the Appendix A, detailing the environment and system layers used to compose the datasets for the City and Highway domains. These tables specify the scenario components, modeling tools, and simulation parameters defining each layer, from physical infrastructure and environmental effects to sensor configurations and control coordination. The scenario set covers low-speed loop driving within urban environments and high-speed driving along highway sections, capturing the ego vehicle’s operation under diverse traffic and environmental conditions. In addition, the scenes from the bus station docking sequence, as detailed in [49,51], are included.

### 5.3. Generated Dataset

**Figure 8:**
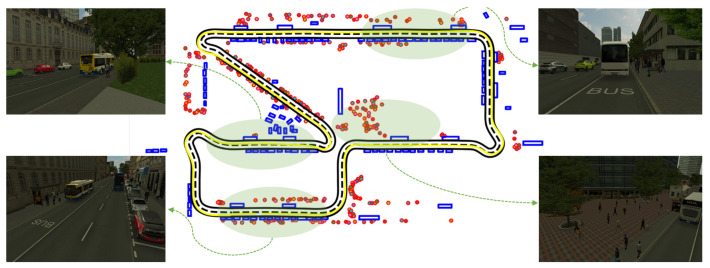
Example of the CityCenter loop scenario with visualized scenes. Blue rectangles indicate vehicles, red circles indicate pedestrians, and green areas indicate bus station zones.

The final dataset consists of multiple driving loops within a virtual urban area (Figure 8 illustrates one loop within the virtual urban area), each covering approximately 6 to 7 bus stations. These loops are organized into 6 data groups, each corresponding to a distinct ego-vehicle trajectory and traffic configuration. In addition to the city groups, 4 highway groups were generated using the same framework to increase scene diversity.

For each group, the reference subset (clear weather) was re-simulated to produce six additional weather-specific subsets by applying controlled visual and physical variations. The subsets correspond to 3 rain conditions (Light Rain (L-Rain), Medium Rain (M-Rain), and Heavy Rain (H-Rain)) and 3 fog conditions (Light Fog (L-Fog), Medium Fog (M-Fog), and Dense Fog (D-Fog)). The configuration of weather-related parameters, including rain intensity, fog density, illumination level, and surface reflectivity, as well as sensor effects such as exposure, noise, and glow, follows the specifications defined in the PRISSMA project deliverable. All data streams were recorded at 25 Hz and a resolution of 1280 × 720. For each group, 15,000 frames were sampled, covering both front and rear camera views. When the dataset is used for training purposes, it is split into 70% for training and 30% for validation. This complete collection is hereafter referred to as ADSceneSim dataset.

**Figure 9:**
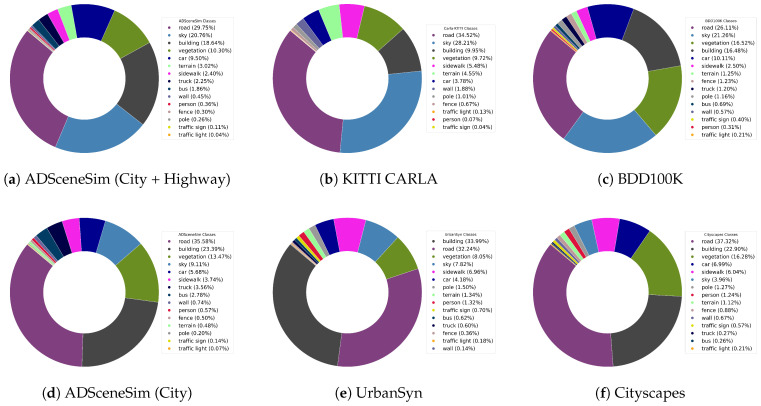
Class-wise pixel distribution across real and synthetic datasets.

The ADSceneSim dataset follows the BDD100K [61] annotation format, providing both semantic and instance-level labels for each image. As shown in the legend of Figure 9, the semantic labels cover 15 categories, while the instance labels include four dynamic objects (car, truck, bus, and pedestrian). Figure 9 compares the class-wise pixel distribution of ADSceneSim with real and synthetic datasets. Compared with synthetic datasets such as UrbanSyn and KITTI-CARLA [62], the ADSceneSim (City + Highway) shows a distribution similar to BDD100K, as both include a mix of urban and highway scenes. In contrast, ADSceneSim (City) is closer to Cityscapes [63], with static categories such as roads and buildings occupying a larger proportion. Overall, ADSceneSim has a comparable class composition to public benchmarks but also shows some differences. For instance, ADSceneSim contains a higher proportion of truck and bus categories, reflecting its higher representation of heavy vehicles and public transport scenarios within the operational domain.

The computational cost of data rendering and collection was also analyzed. Under clear-weather conditions, the simulation and image collection pipeline operated at approximately 22.4 FPS. Fog introduced only minor overhead, reducing the speed by approximately 0.2–0.4 FPS across different densities. In contrast, rain conditions significantly increased the rendering cost due to particle-based effects and reflections, reducing the speed to approximately 0.3 FPS under light rain and below 0.1 FPS under heavy rain. Although rain rendering remains computationally expensive, the process is performed offline, and the generated datasets can be reused for multiple perception evaluation and training tasks.

## 6. Experiments and Evaluation

As described in Section 5, the framework is applied to the selected use case to generate synthetic data. This section aims to evaluate the generated data and its effectiveness for perception model testing and training. First, the data quality is quantified using several fidelity metrics to evaluate how realistically the synthetic data represents real-world conditions relevant to perception model testing and training. Second, we examine, in comparison with real data processed by detection, instance segmentation, and semantic segmentation algorithms, whether such synthetic data can be reliably used to: (1) evaluate public visual perception models trained with real datasets and stress-test the models under adverse conditions rarely encountered in conventional real datasets, and (2) train models to improve robustness under degraded conditions. In this study, weather conditions were selected as the primary factor to assess the robustness of model performance across different domains. Through these experiments, we aim to demonstrate that the proposed framework can generate realistic and task-relevant synthetic data, enabling consistent and reliable evaluation and improvement of visual perception models’ robustness under systematically controlled adversity.

### 6.1. Evaluation Settings

#### 6.1.1. Dataset Description

We evaluate the effectiveness of the synthetic data using two real-world benchmarks, BDD100K and ACDC [64]. BDD100K provides 100,000 driving images captured under diverse conditions, including urban and highway environments, as well as various weather situations such as rain and fog. The instance annotation includes 10 categories, while the semantic segmentation subset (BDD10K) contains 10,000 images annotated with 19 classes, as in Cityscapes. ACDC focuses on adverse visual conditions such as fog, rain, snow, and night, comprising 4006 densely annotated urban images with the same semantic classes as BDD100K and 8 object categories. In addition, we also used SHIFT dataset (100k frames), which focuses on multi-weather synthetic driving scenes, serving as a synthetic benchmark for comparison with ADSceneSim.

#### 6.1.2. Evaluation Metrics

Our demonstration is based on comparing the results of representative perception algorithms for detection and segmentation applied to both synthetic data generated using the proposed method and real-world datasets. Different evaluation metrics are adopted according to each task type. Object detection and instance segmentation are evaluated using COCO-style metrics, including mean Average Precision (mAP) computed over IoU thresholds from 0.5 to 0.95, mAP50 at a fixed 0.5 IoU, and mean Average Recall at top-*K* predictions (mAR1, mAR10, mAR100). Scale-specific performance is further reported as mAPs, mAPm, and mAPl for small (area $<32^{2}$ pixels), medium ($32^{2}\leq$ area $<96^{2}$ pixels), and large (area $\geq 96^{2}$ pixels) objects. For instance segmentation, both box-level (B) and mask-level (M) evaluations are conducted to distinguish spatial localization from pixel-level accuracy. Semantic segmentation is assessed using mean Intersection-over-Union (mIoU) and mean pixel-wise accuracy (mAcc).

#### 6.1.3. Experimental Setup

The simulations are conducted on a workstation equipped with a 16-core Intel i9-12950HX 2.30 GHz CPU and an NVIDIA RTX A4500 GPU. Model training is performed on a high-performance computing server featuring a 32-core Intel Xeon Platinum 8352V 2.10 GHz CPU and 4 NVIDIA RTX 4090 GPUs. Depending on the perception task and training strategy, individual training runs typically required between 4 and 12 h. Ultralytics YOLO [65], OpenMMLab’s MMDetection [66] and MMSegmentation [67] frameworks are employed for the implementation and evaluation of all models used in this paper. Moreover, all experiments are conducted in PyTorch 2.5.1 with CUDA 12.4.

### 6.2. Evaluation of Sensor and Image Fidelity

The physical accuracy of the virtual camera implemented in the Pro-SiVIC^TM^ platform has been experimentally validated, showing a close correspondence with real optical sensors in terms of focal length, distortion, vignetting, and sensor linearity [53]. These models enable the generation of synthetic images that more closely follow realistic sensor behaviour. An important question that arises is how to evaluate whether these data are sufficiently faithful to real-world conditions when used in perception workflows, particularly in relation to the domain gap between synthetic and real images. Evaluating the realism of synthetic images remains challenging because visual realism is influenced by multiple physical and perceptual factors. Therefore, a single visual characteristic is generally insufficient to represent the fidelity of synthetic data with respect to real-world image distributions. In this work, several metrics proposed in [68,69] are adopted to evaluate complementary visual characteristics. These metrics combine texture information using Gray-Level Co-occurrence Matrix (GLCM) [70] and Local Binary Pattern (LBP) [71], as well as high-frequency information with Discrete Cosine Transform (DCT) [72]. These methods produce feature representations that are used as inputs to three convolutional neural networks. These networks determine the level of fidelity of synthetic images through three distinct scores. The fourth score (sH) is derived from GLCM via a linear combination of Haralick metrics.

In this section, we present a comparison of the above-mentioned fidelity metrics between our dataset and several commonly used synthetic datasets. Fidelity score here denotes how closely the visual characteristics of synthetic images resemble those of real scenes. Table 1 reports the computed metrics for the ADSceneSim dataset alongside several synthetic datasets, namely GTA V, Virtual KITTI (vKITTI), KITTI-CARLA, and SYNTHIA, while also including several real-world datasets as references for comparison. Across most metrics, our dataset achieves higher scores than the compared synthetic datasets. In particular, the texture-related metrics, including GLCM, LBP, and sH, show noticeable improvements, whereas the comparatively lower DCT score suggests that the high-frequency characteristics of the generated images could still be further refined. In addition, the relatively high GLCM values highlight the importance of interpreting the four fidelity scores collectively rather than drawing conclusions from individual metrics alone. Overall, these metrics provide complementary indications of the fidelity characteristics of the generated dataset while also revealing remaining differences between synthetic and real-world image distributions.

### 6.3. Evaluation of Model Performance on Synthetic Data

In this section, we evaluate several widely used visual perception models, including Faster R-CNN [73] and FCOS [74] for object detection, Mask R-CNN [75] and GCNet [76] for instance segmentation, and Deeplabv3+ [77] and FCN [78] for semantic segmentation. For fair initialization and comparison, all detection and instance segmentation models use a ResNet-50 backbone with a Feature Pyramid Network (R-50-FPN), whereas semantic segmentation models adopt a dilated ResNet-50 backbone with an output stride of 8 (R-50-D8). All models are initialized with their publicly released weights from the BDD model zoos, with detection models trained on the 100K set and segmentation models trained on the 10K set. The models are then evaluated within the MMDetection and MMSegmentation frameworks on our ADSceneSim dataset, which in this experiment is organized by weather domain as described in Section 5.3. The purpose of this evaluation is to verify whether the datasets generated by the proposed framework can effectively evaluate model performance under controlled degradation, such as adverse weather, which are currently insufficiently represented in existing real-world benchmarks.

The quantitative results in Table 2 and Table 3 show a clear and progressive decline in performance as weather severity increases. For instance, in the segmentation task under fog conditions, Deeplabv3+’s mIoU drops from 65.1 (clear) to 55.9 (dense fog), while mAcc decreases from 80.6 to 78.3. Similarly, under rain conditions, FCN shows a decline in mIoU from 60.8 (clear) to 50.5 (heavy rain), and in mAcc from 77.8 to 68.3. These results confirm that the synthetic dataset produced by our framework introduces realistic visual degradations that effectively stress perception models.

Beyond these overall trends, Table 2 shows that for both detection and instance segmentation algorithms, mAP decreases more in rain, whereas mAP50 decreases more in fog. For example, with Faster R-CNN, mAP drops by about 30% in heavy rain compared with around 25% in dense fog, whereas mAP50 decreases by more than 30% in dense fog relative to about 24% in heavy rain. This distinction is consistent with how mAP and mAP50 are defined: mAP uses multiple IoU thresholds and is therefore more sensitive to localization errors. In contrast, mAP50 relies on a single, lower threshold, so predictions count as correct if the object is detected in approximately the right location, making the metric more influenced by missed detections. As described in [79], rain introduces streaks and droplets that occlude parts of objects and distort their shapes, leading to less accurate bounding-box regression and thus a larger drop in mAP. Fog, on the other hand, reduces contrast and visibility, making distant or small objects appear faint or unclear, which increases missed detections and results in a larger decline in mAP50. The divergence between mAP and mAP50 trends across weather types thus provides further evidence of the realism and evaluation utility of our generated synthetic dataset.

In addition, Table 3 reports the progression of recall metrics: mAR1, mAR10, and mAR100. The results show that mAR1 decreases as weather severity increases across both object detection and instance segmentation, indicating that the highest-confidence predictions become less reliable in degraded conditions. This is because factors such as reduced visibility, blurred contours, and texture corruption (caused by rain or fog) degrade the feature representations that drive confidence scoring, causing high-scoring false positives or true positives that are ranked too low to be included in mAR1. At the same time, the shrinking gains from mAR1 to mAR10 and from mAR10 to mAR100 indicate two observations. First, as weather conditions worsen, models struggle to correctly rank true positives, reducing the incremental recall between the top-1 and top-10 predictions. Second, the quality of lower-ranked predictions also decreases, as reflected by the smaller gains from mAR10 to mAR100. In essence, adverse weather disrupts both confidence estimation and ranking stability, limiting the model’s ability to recover true positives even when more predictions are considered. These trends demonstrate that the synthetic weather variations generated by our framework introduce structured and meaningful visual disturbances, leading to challenges in confidence reliability, ranking robustness, and recall recovery.

### 6.4. Evaluation Under Domain Shift

In the previous section, we demonstrated that our synthetic dataset, ADSceneSim, can effectively stress perception models under controlled adverse conditions, resulting in clear and consistent performance degradation across different weather groups. In this section, we extend our experiments to examine whether the performance trends observed in our synthetic data align with those obtained from a real dataset under similar conditions.

**Figure 10:**
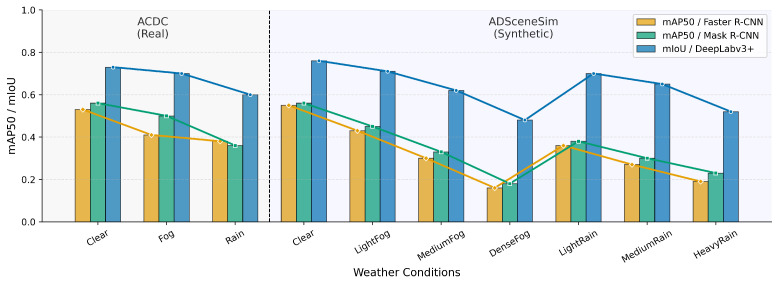
Model performance (trained on clear weather conditions) across weather domains on ACDC and ADSceneSim.

Inspired by the evaluation protocol used in [10], we select 3 models from the previous evaluation section for further analysis. These models are Faster R-CNN for object detection, Mask R-CNN for instance segmentation, and DeepLabv3+ for semantic segmentation. Each model is trained independently using the clear-weather subsets of ACDC (real data) and ADSceneSim (synthetic data) within the MMDetection and MMSegmentation frameworks. The trained models are then evaluated on the corresponding fog and rain subsets of both real and synthetic datasets, allowing us to compare how model performance changes as visual conditions become increasingly challenging. ACDC is used in this section as the benchmark since it provides complete weather annotations across all 3 tasks, whereas BDD does not offer such labels for its segmentation subset (10k set). As shown in Figure 10, the degradation trends across the two datasets follow a similar progression from clear to fog and rain. Although their absolute performance levels slightly differ, both datasets produce comparable relative declines across weather groups. These results confirm the consistency of the synthetic conditions generated by our framework with the real world, supporting the usefulness of ADSceneSim for evaluating perception robustness under adverse conditions.

### 6.5. Evaluation of Model Performance Gains from Synthetic Data

#### 6.5.1. Quantitative Results

In this section, we further investigate whether the synthetic data generated by ADSceneSim can improve model training on real-world perception tasks, rather than being used only for evaluation. Our goal is not to apply domain adaptation techniques, but to directly test whether synthetic data can provide actual training benefits when integrated into real-world perception workflows. We evaluate three widely used public models: YOLOv5 [65] for object detection, YOLOv5-seg for instance segmentation, and DeepLabv3 [80] for semantic segmentation. In addition to ADSceneSim, we also include the SHIFT dataset as a synthetic data baseline, providing a direct comparison with an existing large-scale synthetic benchmark. The ACDC dataset serves as an out-of-distribution benchmark.

For YOLOv5 and its instance-segmentation variant, both implemented in the Ultralytics framework, we follow the default training settings provided by the official repository, with only minor adjustments to batch size and training epochs. For semantic segmentation, DeepLabv3 with a R-50-D8 backbone is trained using MMSegmentation under its standard SGD configuration and common data-augmentation strategies. The contribution of synthetic data is examined through two training strategies. The first strategy follows a transfer learning setup, where the model is pretrained on a synthetic dataset (ADSceneSim or SHIFT) and subsequently fine-tuned on the BDD training set (BDD Train), while the second employs a mixed training scheme that combines synthetic data with BDD Train in a fixed ratio (3:7 in our experiments). All results are compared against a baseline trained only on BDD Train. Model performance is evaluated on two real-world sets: the BDD validation set (BDD Val) and ACDC. While both are real-world datasets, they differ in scene composition, weather diversity, and annotation style, thus introducing a natural domain gap. This setup allows us to evaluate whether the benefits of training with synthetic data can generalize to other real-world driving perception domains. In addition, the performance of the model is reported using standard class-wise metrics, including mAP50 and mIoU.

For the BDD Val, all models are evaluated in-distribution, i.e., within the same domain as BDD Train. In the detection task, both transfer and mixed training improve performance over the BDD-only baseline across most classes (Table 4). When comparing the two synthetic datasets, ADSceneSim tends to produce larger gains than SHIFT for the majority of classes and weather conditions. SHIFT shows higher scores only in a few isolated cases, and the differences are generally small. For both instance and semantic segmentation, weather-specific analysis is not available on the 10k set of BDD Val due to missing related labels, so only overall results are reported. Nevertheless, the advantages of ADSceneSim remain (Table 5 and Table 6), with ADSceneSim outperforming baseline and SHIFT in most categories. On ACDC, the impact of synthetic training also shows clear differences. When using ADSceneSim, both transfer and mixed training improve performance over the BDD-only baseline in most classes and weather conditions, with only a small number of cases showing limited or negative gains. These improvements are particularly evident under rain and fog, which are systematically controlled and captured in the ADSceneSim dataset following its ODD-based design. In contrast, SHIFT leads to negative gains more frequently and sometimes with a larger magnitude, regardless of whether transfer or mixed training is used. These drops are particularly noticeable in detection and instance segmentation under fog and rain, highlighting the domain gap between SHIFT and ACDC.

While ADSceneSim generally yields positive improvements, a few exceptions remain. For example, detection performance for the bus class under fog in ACDC and instance-segmentation performance for the truck class under rain fall below the real-data baseline when using transfer or mixed training. For semantic segmentation, both strategies improve overall IoU, but some categories (such as sidewalk) show inconsistent gains across datasets and weather conditions. These cases suggest that a residual domain gap persists between synthetic and real data, likely caused by differences in appearance details, scene context, and annotation patterns. Despite these limitations, our work demonstrates that structured synthetic datasets, when integrated through transfer or mixed training, can contribute to the enhancement of model robustness under adverse conditions.

#### 6.5.2. Qualitative Comparison

**Figure 11:**
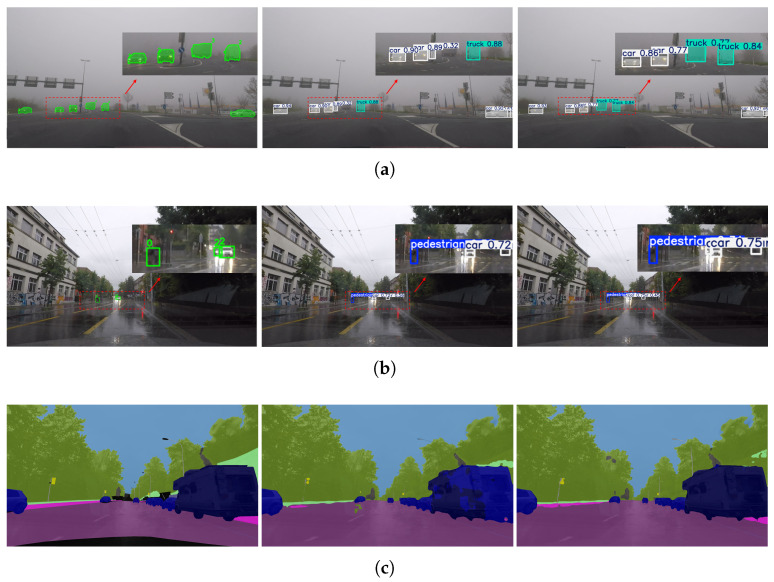
Qualitative comparison on ACDC (mixed training with ADSceneSim + BDD). (**a**) Instance Segmentation (YOLOv5-Seg performing under foggy conditions); (**b**) Object Detection (YOLOv5 performing under rainy conditions); (**c**) Semantic Segmentation (DeepLabv3 performing under rainy conditions).

**Figure 12:**
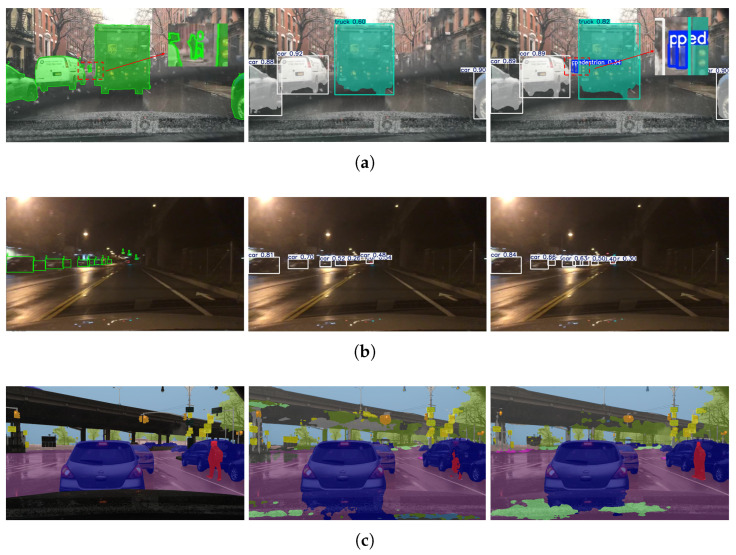
Qualitative comparison on BDD100K Val (pretrained on ADSceneSim → fine-tuned on BDD Train). (**a**) Instance Segmentation (YOLOv5-Seg performing under rainy conditions); (**b**) Object Detection (YOLOv5 performing under night-foggy conditions); (**c**) Semantic Segmentation (DeepLabv3 performing under rainy conditions).

In addition to quantitative evaluations, Figure 11 and Figure 12 provide a qualitative demonstration of visual perception results under challenging weather conditions on the ACDC and BDD val datasets. Each row shows three views for the same scene: the ground-truth annotation (left), the prediction from a model trained only on BDD (middle), and the prediction from a model trained with ADSceneSim and BDD (right).

From the visualized prediction of ACDC dataset (Figure 11), we can observe that models trained with mixed data show robust instance segmentation of objects under fog (specifically, recovering fog-obscured trucks missed by the baseline), and improved detection of distant targets such as vehicles in rainy scenes. For semantic segmentation, synthetic-enhanced models produce more complete and accurate predictions, especially for vehicles and sidewalks, correcting the fragmented predictions seen in the baseline. On BDD val (Figure 12), similar improvements are observed. Under rainy and night-fog conditions, synthetic-pretrained models recover distant or low-contrast objects missed by BDD-only models, and further demonstrate improved semantic segmentation quality with more coherent masks, particularly for foreground objects such as vehicles and pedestrians.

### 6.6. Ablation Study on Framework Components

An ablation study is further conducted on the instance segmentation task using YOLOv5-seg model following the configuration of mixed training strategy described in Section 6.5. The study evaluates the contribution of different components within the proposed data generation framework, including operational conditions specified through ODD and OEDR descriptions, the introduction of adverse conditions, and the proposed visibility-aware annotation strategy.

Table 7 presents the performance obtained by progressively enabling different components during the generation of the ADSceneSim synthetic dataset using the proposed framework. Starting with a baseline model trained only on BDD100K, ADSceneSim datasets with increasingly complete configurations are progressively incorporated during the training phase. The configuration considering only operational conditions already improves performance across all benchmarks, including the BDD validation set and ACDC weather subsets. Compared with the baseline, the model achieves gains of +2.2 Box mAP50 and +2.3 Mask mAP50 on BDD val, while also showing improvements under adverse conditions, particularly on ACDC Rain. These results suggest that organizing synthetic data according to operational conditions helps generate training samples that better reflect the target driving scenarios and environmental variations encountered during evaluation.

When adverse conditions (weather) are further introduced, performance continues to increase across all datasets. The largest gains are observed under foggy and rainy conditions, where the additional weather-aware synthetic data provides better coverage of visibility degradation and environmental perturbations. For example, the Box mAP50 on ACDC Rain increases from 30.6 to 33.1, while the Mask mAP50 improves from 27.9 to 30.2. Finally, enabling the proposed visibility-aware annotation strategy further improves instance segmentation performance, particularly under degraded conditions where object visibility is significantly affected by adverse weather. Compared with the previous configuration, the final model achieves an additional gain of +2.1 Box mAP50 and +1.9 Mask mAP50 on ACDC Rain. Overall, the results support the effectiveness of the proposed framework components, with progressively improved instance segmentation performance observed as additional components are introduced.

### 6.7. Discussion and Limitations

Despite the encouraging results obtained with the proposed framework and the generated ADSceneSim dataset, several limitations remain and should be discussed.

First, although the generated dataset ADSceneSim demonstrates relatively higher fidelity according to multiple metrics and produces representative degradations for perception evaluation, our synthetic environments still cannot fully reproduce the complexity and variability of real-world driving conditions. It is still challenging to accurately reproduce certain physical phenomena in simulation, such as irregular reflections, illumination transitions, sensor contamination, and complex atmospheric interactions. In addition, a domain gap between synthetic and real data is still observed during model training, indicating that synthetic data cannot completely replace real-world data. Consequently, the generated data should be viewed as a complementary resource for perception evaluation, robustness analysis, and training support alongside real-world testing.

Second, the current implementation focuses primarily on fog and rain conditions under different illumination settings. While these conditions represent important adverse factors affecting visual perception systems, other degradations, including snow, icy roads, severe glare, and extreme low-visibility situations, are not yet covered. Extending the framework toward a broader range of environmental conditions and sensor degradations, therefore, remains an important direction for future work.

Third, the proposed framework adopts a requirement-driven generation strategy guided by ODD and OEDR specifications derived from targeted autonomous driving use cases. As a result, the generated scenarios are intentionally constrained to operationally relevant conditions rather than aiming at unconstrained visual diversity. While this improves traceability and controllability during evaluation, the resulting dataset coverage remains influenced by the selected operational domains and use cases. This limitation could be mitigated through the inclusion of additional use cases and broader operational conditions.

## 7. Conclusions and Future Work

This work presented a scenario-driven synthetic data generation framework designed to support the evaluation of visual perception functions under adverse driving conditions. By using ODD specifications and OEDR-derived perception requirements obtained from the target use cases as guiding elements, the framework structures scenario creation into environment and system layers, enabling controlled variation of environmental, behavioral, and system factors. Across its three stages of scenario configuration, simulation-based data generation, and annotation post-processing, the pipeline provides a modular and semi-automatic workflow that can be adapted to diverse autonomous-driving use cases. Its implementation in Pro-SiVIC^TM^ and RTMaps^TM^ demonstrates the practical applicability of the framework and offers insight into its potential portability to other platforms and toolchains.

Through the application to use cases from PRISSMA and AUGMENTED CCAM projects, we generated the ADSceneSim dataset and performed a comprehensive evaluation of its fidelity and utility. Experimental results confirm several key findings. First, the generated dataset ADSceneSim shows encouraging fidelity not only in visual appearance but also in semantic distributions. As illustrated in Figure 9, the class-wise pixel distribution of ADSceneSim follows trends similar to those observed in real-world datasets, indicating a reasonable semantic composition at the dataset level. Second, structured adverse scenarios were shown to introduce predictable and measurable degradations in perception models, demonstrating the capacity of the synthetic data to simulate real-world visual challenges in a controlled manner. Finally, experiments with transfer learning and mixed training strategies indicate that, although a domain gap remains, the synthetic data can still contribute to improved robustness and generalization of deep learning models across diverse conditions. Overall, these findings demonstrate the effectiveness of the proposed scenario-driven framework, showing that the synthetic datasets it produces can serve as a practical complement to real-world benchmarks for repeatable, configurable, and safety-aligned perception evaluation.

Future work will extend the proposed framework along several directions. First, integrating OpenDRIVE, OpenSCENARIO, and OpenLABEL will enhance interoperability and facilitate deployment across different simulation tools and industrial toolchains. Second, further improving visual realism through physically based rendering, domain-adaptation techniques, and generative models will help reduce the remaining synthetic–real domain gap and increase the external validity of the generated data. Third, the framework can be applied to additional use cases to generate datasets that capture a broader range of traffic interactions, multi-agent dynamics, and adverse conditions, thereby extending its applicability across diverse operational domains. Finally, extending the framework to multimodal sensing, including LiDAR, Radar, would broaden its applicability to sensor-fusion pipelines. Combining the synthetic data generation process with more high-fidelity digital twins of real-world environments represents a promising direction, as it would enable the creation of more realistic and context-accurate datasets that better reflect real-world operating environments.

## Figures and Tables

**Table 1:** Fidelity scores (%) on synthetic and real datasets, with best results in bold and second best underlined (on synthetic).

Fidelity Metrics	Synthetic Datasets					Real-World Datasets			
	ADSceneSim (Ours)	GTA V	Virtual KITTI	KITTI CARLA	SYNTHIA	Cityscapes	KITTI	NuScenes	ONCE
GLCM	**89.5**	12.0	0.05	0.4	10.4	96.7	99.8	90.9	97.1
LBP	**32.4**	19.1	2.2	5.9	22.5	87.5	94.9	60.5	63.3
DCT	22.8	4.3	**51.2**	1.6	3.6	98.1	96.9	98.5	76.1
sH	**50.3**	48.1	34.1	39.0	41.8	72.8	62.4	73.6	72.7

**Table 2:** Performance comparison of detection, instance segmentation, and semantic segmentation models on ADSceneSim.

Algorithm	Metric	BDD Val	Clear	L-Rain	M-Rain	H-Rain	L-Fog	M-Fog	D-Fog
Faster R-CNN	mAP/mAP50	32.3/57.5	37.7/56.9	32.7/51.0	29.7/47.4	26.3/43.4	37.6/56.3	32.0/47.6	27.0/38.7
	mAP(s/m/l)	15.7/37.5/52.8	15.5/53.9/71.3	11.9/46.5/67.7	10.2/42.5/63.7	8.6/37.6/59.2	14.7/49.5/71.0	10.5/45.9/70.2	6.2/38.6/67.7
	mAR(s/m/l)	26.5/48.0/60.7	20.9/65.1/79.6	16.4/60.0/78.2	14.2/56.5/76.0	12.3/51.0/72.0	19.7/59.6/79.2	13.2/55.9/78.3	7.3/45.1/76.5
FCOS	mAP/mAP50	30.6/55.3	36.3/54.8	30.9/48.3	28.0/44.8	25.2/41.9	34.1/51.5	32.6/48.7	28.5/41.8
	mAP(s/m/l)	13.4/35.6/51.0	13.4/52.5/73.2	9.9/44.1/68.3	8.5/39.8/63.9	7.4/35.7/59.5	11.3/49.0/72.2	10.0/46.9/71.4	6.4/41.0/69.7
	mAR(s/m/l)	25.9/48.8/61.2	21.1/66.8/82.5	15.9/60.9/80.1	13.5/56.7/77.6	12.0/52.3/73.4	18.5/62.9/81.8	15.6/61.4/81.3	9.0/55.9/80.5
Mask R-CNN	mAP/mAP50	19.9/35.2	20.2/31.5	17.3/27.7	14.2/24.0	11.4/20.1	19.2/29.7	17.6/26.9	14.5/21.0
	mAP(s/m/l)	10.2/24.9/43.1	9.0/34.8/51.2	6.7/30.6/50.5	5.4/25.1/43.9	4.2/20.5/35.4	7.7/33.8/51.5	6.3/32.2/50.8	3.6/28.6/48.9
	mAR(s/m/l)	14.8/30.0/46.5	13.4/40.9/55.3	10.6/37.1/54.9	8.8/32.3/50.2	7.0/26.9/42.9	11.4/40.2/55.3	9.4/37.9/54.5	4.6/33.4/52.9
GCNet	mAP50/mAP	20.0/36.3	20.5/32.0	17.3/27.6	14.4/23.9	12.0/20.8	18.9/29.4	17.4/27.5	15.0/23.3
	mAP(s/m/l)	10.3/26.4/36.8	9.0/34.9/51.8	6.5/30.2/48.5	5.3/25.2/41.4	4.5/21.0/34.5	7.6/32.6/50.7	6.4/31.0/49.1	4.8/28.2/47.3
	mAR(s/m/l)	14.6/32.1/40.8	13.8/41.3/56.8	10.5/37.1/56.2	8.7/32.4/50.6	7.4/28.1/43.6	11.1/40.6/56.9	10.0/38.2/54.7	7.1/35.0/52.1
FCN	mIoU	59.9	60.8	57.8	54.4	50.5	60.2	56.2	51.1
	mAcc	74.3	77.8	74.8	71.8	68.3	76.8	74.2	71.0
Deeplabv3+	mIoU	61.6	65.1	60.9	58.3	54.4	64.4	60.8	55.9
	mAcc	75.1	80.6	76.7	74.3	70.5	80.5	79.5	78.3

**Table 3:** mAR across 1, 10, and 100 proposals for detection and instance segmentation models on ADSceneSim.

Algorithm	Backbone	Metric	BDD Val	Clear	L-Rain	M-Rain	H-Rain	L-Fog	M-Fog	D-Fog
Faster R-CNN	R-50-FPN	mAR1	22.1	17.6	16.1	15.1	13.9	18.7	15.6	13.4
		mAR10 ($\Delta_{10-1}$) ^a^	40.0 (+17.9)	42.8 (+25.1)	38.8 (+22.7)	36.2 (+21.1)	33.0 (+19.1)	43.0 (+24.3)	36.5 (+20.9)	29.9 (+16.5)
		mAR100 ($\Delta_{100-10}$) ^b^	41.8 (+1.8)	44.5 (+1.7)	40.3 (+1.5)	37.6 (+1.4)	34.2 (+1.2)	44.5 (+1.5)	37.6 (+1.2)	30.7 (+0.9)
FCOS	R-50-FPN	mAR1	21.2	17.3	15.8	14.7	13.7	16.4	15.8	14.5
		mAR10 ($\Delta_{10-1}$)	40.3 (+19.1)	43.5 (+26.2)	38.8 (+23.0)	35.8 (+21.1)	33.1 (+19.4)	41.1 (+24.7)	39.2 (+23.4)	34.3 (+19.8)
		mAR100 ($\Delta_{100-10}$)	42.5 (+1.8)	45.3 (+1.8)	40.3 (+1.5)	37.4 (+1.6)	34.5 (+1.4)	42.9 (+1.8)	40.9 (+1.7)	35.7 (+1.4)
Mask R-CNN	R-50-FPN	mAR1	14.3	13.0	11.5	10.5	8.8	12.4	11.8	10.1
		mAR10 ($\Delta_{10-1}$)	23.3 (+9.0)	29.6 (+16.6)	25.8 (+14.3)	22.7 (+12.2)	18.9 (+10.1)	27.1 (+14.7)	25.0 (+13.2)	20.9 (+10.8)
		mAR100 ($\Delta_{100-10}$)	24.2 (+0.9)	30.3 (+0.7)	26.2 (+0.4)	23.0 (+0.3)	19.1 (+0.2)	27.6 (+0.5)	25.4 (+0.4)	21.2 (+0.3)
GCNet	R-50-FPN	mAR1	14.8	13.5	11.8	10.3	9.0	12.5	11.8	10.1
		mAR10 ($\Delta_{10-1}$)	23.7 (+8.9)	30.6 (+17.1)	26.0 (+14.2)	22.7 (+12.4)	19.4 (+10.4)	27.3 (+14.8)	25.6 (+13.8)	22.1 (+12.0)
		mAR100 ($\Delta_{100-10}$)	24.6 (+0.9)	31.3 (+0.7)	26.5 (+0.5)	23.0 (+0.3)	19.7 (+0.3)	27.9 (+0.6)	26.0 (+0.4)	22.4 (+0.3)

^a^ $\left(\Delta_{10-1}\right)$ denotes the gain between mAR10 and mAR1. ^b^ $\left(\Delta_{100-10}\right)$ denotes the gain between mAR100 and mAR10.

**Table 4:** Detection (YOLOv5) evaluation on BDD100k and ACDC datasets across training strategies and weather conditions.

Training Strategy	Training Dataset	Weather	BDD Val (Bounding Box AP50 ($\Delta_{B}$ ^a^))				ACDC (Bounding Box AP50 ($\Delta_{B}$ ^a^))			
			Car	Person	Bus	Truck	Car	Person	Bus	Truck
Baseline	BDD	Clear	83.9	62.8	57.4	58.6	76.0	52.5	34.7	54.8
Transfer	ADSceneSim → BDD	Clear	83.9 (+0.0)	63.2 (+0.4)	59.1 (+1.7)	59.3 (+0.7)	76.1 (+0.1)	53.4 (+0.9)	**35.9** ^b^ (+1.2)	53.8 (−1.0)
	SHIFT → BDD	Clear	83.2 (−0.7)	59.4 (−3.4)	56.9 (−0.5)	57.7 (−0.9)	76.1 (+0.1)	53.5 (+1.0)	32.7 (−2.0)	51.7 (−3.1)
Mixed	ADSceneSim+BDD	Clear	83.9 (+0.0)	**63.9** (+1.1)	**62.1** (+4.7)	**60.7** (+2.1)	**76.5** (+0.5)	**53.5** (+1.0)	34.5 (−0.2)	**56.0** (+1.2)
	SHIFT+BDD	Clear	**84.0** (+0.1)	63.4 (+0.6)	58.0 (+0.6)	59.5 (+0.9)	75.8 (−0.2)	53.0 (+0.5)	32.9 (−1.8)	55.4 (+0.6)
Baseline	BDD	Rain	80.7	59.0	59.7	57.9	**86.0**	51.5	40.1	**46.8**
Transfer	ADSceneSim → BDD	Rain	**80.9** (+0.2)	**60.0** (+1.0)	**61.0** (+1.3)	58.2 (+0.3)	85.5 (−0.5)	51.5 (+0.0)	38.2 (−1.9)	46.0 (−0.8)
	SHIFT → BDD	Rain	79.8 (−0.9)	55.8 (−3.2)	56.6 (−3.1)	58.0 (+0.1)	85.1 (−0.9)	50.7 (−0.8)	33.6 (−6.5)	43.6 (−3.2)
Mixed	ADSceneSim+BDD	Rain	80.7 (+0.0)	59.7 (+0.7)	60.3 (+0.6)	58.8 (+0.9)	85.9 (−0.1)	**52.8** (+1.3)	**40.1** (+0.0)	44.0 (−2.8)
	SHIFT+BDD	Rain	80.2 (−0.5)	59.6 (+0.6)	57.4 (−2.3)	**62.3** (+4.4)	85.9 (−0.1)	51.3 (−0.2)	36.2 (−3.9)	44.6 (−2.2)
Baseline	BDD	Fog	80.6	50.8	**84.3**	44.4	85.9	70.2	66.4	71.8
Transfer	ADSceneSim → BDD	Fog	80.3 (−0.3)	**54.6** (+3.8)	79.5 (−4.8)	44.6 (+0.2)	**86.1** (+0.2)	69.8 (−0.4)	67.4 (+1.0)	71.8 (+0.0)
	SHIFT → BDD	Fog	79.7 (−0.9)	51.8 (+1.0)	84.1 (−0.2)	41.7 (−2.7)	85.6 (−0.3)	70.0 (−0.2)	56.4 (−10.0)	71.4 (−0.4)
Mixed	ADSceneSim+BDD	Fog	**81.0** (+0.4)	50.5 (−0.3)	83.1 (−1.2)	**47.8** (+3.4)	85.8 (−0.1)	**70.2** (+0.0)	**72.4** (+6.0)	**72.7** (+0.9)
	SHIFT+BDD	Fog	80.2 (−0.4)	53.6 (+2.8)	80.8 (−3.5)	42.5 (−1.9)	85.6 (−0.3)	67.0 (−3.2)	60.5 (−5.9)	72.2 (+0.4)

^a^ $\Delta_{B}$ is the gain over baseline; ^b^ Bold values indicate the best result for each class and validation setting.

**Table 5:** Instance segmentation (YOLOv5-seg) evaluation on BDD10k and ACDC across training strategies and weather conditions.

Validation	Training Strategy	Training Dataset	Weather	Bounding Box AP50 ($\Delta_{B}$ ^a^)				Instance Mask AP50 ($\Delta_{B}$ ^a^)			
				Car	Person	Bus	Truck	Car	Person	Bus	Truck
BDD val	Baseline	BDD	All	75.3	57.9	25.0	30.7	67.2	52.5	24.8	30.1
	Transfer	ADSceneSim → BDD	All	75.5 (+0.2)	60.6 (+2.7)	24.9 (−0.1)	32.2 (+1.5)	67.2 (+0.0)	55.6 (+3.1)	25.0 (+0.2)	31.9 (+1.8)
		SHIFT → BDD	All	73.4 (−1.9)	55.2 (−2.7)	20.1 (−4.9)	25.6 (−5.1)	65.1 (−2.1)	49.9 (−2.6)	19.2 (−5.6)	25.7 (−4.4)
	Mixed	ADSceneSim+BDD	All	**76.7** ^b^ (+1.4)	**64.2** (+6.3)	**35.6** (+10.6)	**37.2** (+6.5)	**69.2** (+2.0)	**59.1** (+6.6)	**35.0** (+10.2)	**35.9** (+5.8)
		SHIFT+BDD	All	75.6 (+0.3)	59.4 (+1.5)	27.8 (+2.8)	31.4 (+0.7)	67.2 (+0.0)	54.4 (+1.9)	29.3 (+4.5)	29.3 (−0.8)
ACDC	Baseline	BDD	Clear	70.2	41.9	20.6	38.3	58.9	33.8	20.5	34.9
	Transfer	ADSceneSim → BDD	Clear	69.4 (−0.8)	45.6 (+3.7)	**30.6** (+10.0)	41.9 (+3.6)	59.8 (+0.9)	35.6 (+1.8)	19.2 (−1.3)	33.2 (−1.7)
		SHIFT → BDD	Clear	69.3 (−0.9)	41.5 (−0.4)	15.3 (−5.3)	31.3 (−7.0)	58.3 (−0.6)	31.7 (−2.1)	15.5 (−5.0)	27.4 (−7.5)
	Mixed	ADSceneSim+BDD	Clear	**70.8** (+0.6)	**47.8** (+5.9)	25.4 (+4.8)	**44.5** (+6.2)	**60.7** (+1.8)	**38.3** (+4.5)	**24.1** (+3.6)	**39.3** (+4.4)
		SHIFT+BDD	Clear	69.3 (−0.9)	43.3 (+1.4)	18.7 (−1.9)	38.1 (−0.2)	58.9 (+0.0)	32.5 (−1.3)	17.7 (−2.8)	33.4 (−1.5)
	Baseline	BDD	Rain	79.3	37.4	12.1	**24.2**	72.3	29.2	25.3	23.6
	Transfer	ADSceneSim → BDD	Rain	79.6 (+0.3)	39.5 (+2.1)	11.8 (−0.3)	22.6 (−1.6)	72.4 (+0.1)	33.9 (+4.7)	25.8 (+0.5)	18.2 (−5.4)
		SHIFT → BDD	Rain	78.3 (−1.0)	30.5 (−6.9)	10.9 (−1.2)	8.3 (−15.9)	71.2 (−1.1)	25.0 (−4.2)	20.2 (−5.1)	8.7 (−14.9)
	Mixed	ADSceneSim+BDD	Rain	**81.4** (+2.1)	**44.2** (+6.8)	**21.2** (+9.1)	23.7 (−0.5)	**74.4** (+2.1)	**35.9** (+6.7)	**31.9** (+6.6)	**23.8** (+0.2)
		SHIFT+BDD	Rain	79.8 (+0.5)	33.9 (−3.5)	11.1 (−1.0)	13.4 (−10.8)	72.6 (+0.3)	30.4 (+1.2)	23.0 (−2.3)	13.0 (−10.6)
	Baseline	BDD	Fog	81.5	56.5	40.7	52.7	71.0	45.4	40.5	49.6
	Transfer	ADSceneSim → BDD	Fog	81.6 (+0.1)	**60.0** (+3.5)	**47.5** (+6.8)	52.8 (+0.1)	71.4 (+0.4)	**49.2** (+3.8)	**47.2** (+6.7)	49.5 (−0.1)
		SHIFT → BDD	Fog	79.7 (−1.8)	56.4 (−0.1)	34.6 (−6.1)	42.7 (−10.0)	69.6 (−1.4)	46.6 (+1.2)	33.1 (−7.4)	40.7 (−8.9)
	Mixed	ADSceneSim+BDD	Fog	**82.7** (+1.2)	59.6 (+3.1)	40.1 (−0.6)	**57.4** (+4.7)	**72.3** (+1.3)	48.0 (+2.6)	40.0 (−0.5)	**54.5** (+4.9)
		SHIFT+BDD	Fog	79.3 (−2.2)	50.4 (−6.1)	22.2 (−18.5)	47.0 (−5.7)	71.2 (+0.2)	40.8 (−4.6)	22.1 (−18.4)	46.1 (−3.5)

^a^ $\Delta_{B}$ is the gain over baseline; ^b^ Bold values indicate the best result for each class and validation setting.

**Table 6:** Semantic segmentation (Deeplabv3) evaluation on BDD10k and ACDC across training strategies and weather conditions.

Validation	Training Strategy	Training Dataset	Weather	IoU ($\Delta_{B}$ ^a^)								
				Car	Person	Bus	Truck	Road	Sidewalk	T.Sign	T.Light	Others
BDD val	Baseline	BDD	All	90.5	64.0	77.9	57.8	94.8	66.7	57.2	60.0	55.5
	Transfer	ADSceneSim → BDD	All	91.0 (+0.5)	66.4 (+2.4)	**83.5** ^b^ (+5.6)	**58.3** (+0.5)	95.1 (+0.3)	67.6 (+0.9)	60.9 (+3.7)	62.4 (+2.4)	57.2 (+1.7)
		SHIFT → BDD	All	90.8 (+0.3)	64.2 (+0.2)	79.3 (+1.4)	57.3 (−0.5)	95.1 (+0.3)	65.7 (−1.0)	57.5 (+0.3)	58.1 (−1.9)	50.5 (−5.0)
	Mixed	ADSceneSim+BDD	All	**91.1** (+0.6)	**66.7** (+2.7)	82.4 (+4.5)	58.0 (+0.2)	**95.1** (+0.3)	**68.0** (+1.3)	**61.7** (+4.5)	**62.7** (+2.7)	**58.1** (+2.6)
		SHIFT+BDD	All	90.4 (−0.1)	64.8 (+0.8)	78.4 (+0.5)	56.8 (−1.0)	95.0 (+0.2)	67.8 (+1.1)	58.3 (+1.1)	60.8 (+0.8)	53.7 (−1.8)
ACDC	Baseline	BDD	Clear	83.2	54.5	24.7	59.0	90.8	**63.7**	57.1	58.3	54.1
	Transfer	ADSceneSim → BDD	Clear	82.4 (−0.8)	**59.0** (+4.5)	**26.8** (+2.1)	**63.6** (+4.6)	90.1 (−0.7)	62.4 (−1.3)	**58.3** (+1.2)	**58.7** (+0.4)	**56.6** (+2.5)
		SHIFT → BDD	Clear	84.7 (+1.5)	52.3 (−2.2)	22.5 (−2.2)	63.5 (+4.5)	90.0 (−0.8)	63.0 (−0.7)	52.6 (−4.5)	46.2 (−12.1)	47.6 (−6.5)
	Mixed	ADSceneSim+BDD	Clear	**86.0** (+2.8)	58.9 (+4.4)	26.5 (+1.8)	60.5 (+1.5)	**90.9** (+0.1)	62.8 (−0.9)	57.6 (+0.5)	55.9 (−2.4)	54.9 (+0.8)
		SHIFT+BDD	Clear	82.6 (−0.6)	53.3 (−1.2)	22.2 (−2.5)	47.3 (−11.7)	90.3 (−0.5)	61.2 (−2.5)	57.3 (+0.2)	58.7 (+0.4)	44.7 (−9.4)
	Baseline	BDD	Rain	83.4	47.5	**55.6**	24.2	84.3	48.3	56.0	58.6	51.3
	Transfer	ADSceneSim → BDD	Rain	**86.0** (+2.6)	**54.0** (+6.5)	46.0 (−9.6)	33.2 (+9.0)	**86.4** (+2.1)	**57.0** (+8.7)	**57.5** (+1.5)	**61.0** (+2.4)	**56.4** (+5.1)
		SHIFT → BDD	Rain	82.2 (−1.2)	39.9 (−7.6)	36.7 (−18.9)	28.8 (+4.6)	86.4 (+2.1)	56.1 (+7.8)	51.9 (−4.1)	50.7 (−7.9)	47.9 (−3.4)
	Mixed	ADSceneSim+BDD	Rain	85.7 (+2.3)	50.8 (+3.3)	51.6 (−4.0)	**33.8** (+9.6)	86.0 (+1.7)	55.8 (+7.5)	56.8 (+0.8)	57.4 (−1.2)	51.9 (+0.6)
		SHIFT+BDD	Rain	81.5 (−1.9)	42.9 (−4.6)	44.4 (−11.2)	23.7 (−0.5)	85.8 (+1.5)	55.0 (+6.7)	56.3 (+0.3)	59.5 (+0.9)	46.4 (−4.9)
	Baseline	BDD	Fog	87.5	57.0	46.6	69.3	93.6	64.0	66.0	42.7	49.8
	Transfer	ADSceneSim → BDD	Fog	88.4 (+0.9)	**59.3** (+2.3)	47.8 (+1.2)	**74.5** (+5.2)	93.4 (−0.2)	**64.4** (+0.4)	69.3 (+3.3)	**47.4** (+4.7)	**52.5** (+2.7)
		SHIFT → BDD	Fog	87.0 (−0.5)	49.4 (−7.6)	47.5 (+0.9)	72.3 (+3.0)	**94.2** (+0.6)	62.4 (−1.6)	64.9 (−1.1)	40.1 (−2.6)	49.9 (+0.1)
	Mixed	ADSceneSim+BDD	Fog	**89.6** (+2.1)	57.0 (+0.0)	**52.4** (+5.8)	72.5 (+3.2)	93.5 (−0.1)	63.0 (−1.0)	**69.5** (+3.5)	44.5 (+1.8)	51.0 (+1.2)
		SHIFT+BDD	Fog	83.1 (−4.4)	48.6 (−8.4)	40.6 (−6.0)	61.5 (−7.8)	92.5 (−1.1)	58.4 (−5.6)	68.3 (+2.3)	47.0 (+4.3)	46.5 (−3.3)

^a^ $\Delta_{B}$ is the gain over baseline; ^b^ Bold values indicate the best result for each class and validation setting.

**Table 7:** Ablation study on the main components of the proposed framework for instance segmentation using YOLOv5-seg. Values in parentheses indicate mAP50 gains over the previous configuration.

Training Resources	Synthetic Dataset Configuration			BDD Val		ACDC Clear		ACDC Fog		ACDC Rain	
	ODD/OEDR	Weathers	Re-Annotation	Box	Mask	Box	Mask	Box	Mask	Box	Mask
Baseline BDD	× ^c^	×	×	36.3	32.1	35.1	27.5	48.9	41.5	27.8	25.7
BDD + ADSceneSim (w/Op. conditions ^a^)	✓ ^c^	×	×	38.5 (+2.2)	34.4 (+2.3)	36.2 (+1.1)	28.9 (+1.4)	50.4 (+1.5)	43.3 (+1.8)	30.6 (+2.8)	27.9 (+2.2)
BDD + ADSceneSim (w/Adverse conditions)	✓	✓	×	41.5 (+3.0)	36.7 (+2.3)	38.1 (+1.9)	30.6 (+1.7)	51.4 (+1.0)	44.5 (+1.2)	33.1 (+2.5)	30.2 (+2.3)
BDD + ADSceneSim (w/Visibility ann. ^b^)	✓	✓	✓	43.2 (+1.7)	38.5 (+1.8)	39.9 (+1.8)	32.6 (+2.0)	52.1 (+0.7)	45.4 (+0.9)	35.2 (+2.1)	32.1 (+1.9)

^a^ Operational conditions; ^b^ Visibility-aware annotation; ^c^ ✓ indicates inclusion of the corresponding component, while × indicates its exclusion.

## Data Availability

The raw data supporting the conclusions of this article will be made available by the authors on request.

## Abbreviations

The following abbreviations are used in this manuscript:

ADS	Automated Driving System
DCT	Discrete Cosine Transform
DDS	Data Distribution Service
DDT	Dynamic Driving Task
FIFO	First In First Out
GLCM	Gray-Level Co-occurrence Matrix
HDR	High Dynamic Range
LBP	Local Binary Pattern
ODD	Operational Design Domain
OEDR	Object and Event Detection and Response
SSIM	Structural Similarity
TTC	Time To Collision

## Appendix A

**Table A1:** Detailed configuration of environmental layers in the scenario model.

Dataset	Layers	Classes	Intra-Class Variations	Modeling	Assets or Tools	Textures or Settings
ADSceneSim City (C) Highway (H)	L01	Lane configuration	6 (C) + 4 (H)	ROAD [81]	2 types of roads (urban layout: straight, intersection, T-junction, Y-junction, roundabout, bus pull-in; highway layout: straight, merge, diverge, bridge)	2 (asphalt, concrete)
		Lane marking	5 (C) + 2 (H)	GRoTex [82]	Urban: 1/2/3-way markings (solid/dashed), crosswalks, parking markings, bus lane markings, turn arrows, stop line; Highway: 3-way markings (solid/dashed), merging/diverging markings	1 per type (with random degradation provided by GRoTex)
		Traffic signs and lights	8 (C) + 5 (H)	Int. 3D models ^b^ + Path Edit	Signs: 10 Plaques + 3 pole; Lights: 1 overhead and 1 side-post	1 per sign plaque (more than 20 types), 3 per light, 1 per pole
		Sidewalks	4 (C)	ROAD	1 sidewalk along roads, 2 pedestrain pathway, 3 urban plaza	1 per class (concrete, bricks, gravel, etc.)
	L02	Buildings	55 (C) + 1 (H)	Cust. 3D models ^a^	21 residence, 17 commerce, 5 instituation, 2 landmark, 1 recreation, 1 highway bridge	1 per background building, 2 to 4 per regular building
		Bus stations	8 (C)	Cust. 3D models ^a^	8 different stations	1 for each sub-component of the station: structure, advertisement, sign, light, and seats.
		Vegetation	6 (C) + 3 (H)	Cust. 3D models ^a^	5 trees, 4 terrains	2 per tree, 1 per terrain
		Fence, wall, guardrails	3 (C) + 2 (H)	Cust. 3D models ^a^	4 walls, 2 fences 1 guardrail	1 per fence/wall/guardrail
		Street lamps	2 (C) + 1 (H)	Int. 3D models ^b^	3 street lamps	2 per lamp, 1 per pole
	L03	Temporal signs	2 (C)	Int. 3D models ^b^	2 signs, 2 barriers	1 per sign or barrier, 1 per pole
		Roadwork	1 (C)	Int. 3D models ^b^	1 cone, 1 construction equipment, material, and building	1 to 2 per subcomponent of roadwork area
	L04	Car, truck, van, bus, pickup	121 (C) + 73 (H)	Cust. 3D models ^a^	Static vehicle resources: 15 trucks, 10 vans, 15 buses, 81 cars; Dynamic vehicles (derived from static): 8 groups with ∼10 vehicles each	∼2 per dynamic vehicle (wheels and body), mostly 1 per static vehicle
		Pedestrian	30 (C)	Procedural crowds ^c^	24 groups (15 walking, 2 running, 7 standing), each with ∼5–20 persons	∼4 parts per person (e.g., head, hands, shirt, pants); optional items include hat, sunglasses; clothes in random colors
	L05	Sky	5 (C & H)	6 camera captures in Blender	8 HDRIs ^d^ contains clearblue, partly cloudy, and overcast	6 surfaces per skybox
		Weather conditions	7 (C & H)	Pro-SiVIC^TM^	Rain: simulated particles and dynamic raindrop (on windsheid) filter applied on camera; Fog: homogeneous fog filter applied on camera	3 levels of particles (number, speed, color and range); 3 levels of raindrop (number, size and deformation factor); 3 levels of fog (luminosity and density)
		Illumination variability	Following weather settings	Pro-SiVIC^TM^	Lighting rendering: ambient lighting, positional lighting, shadows (projected, ambient occlusion)	Global ambient settings (enviroment); light source settings (sun position, intensity, color); per-object shadow maps and ambient occlusion maps
		Reflection effect	2 (C & H)	Pro-SiVIC^TM^	2 mechanisms: planar reflection and cube map reflection	Pavement (wet) reflection and car body reflection

^a^ Custom 3D models (.fbx/.obj/.mtl), imported into Blender for usable Pro-SiVIC^TM^ material and texture generation. ^b^ Internal 3D models present in Pro-SiVIC^TM^. ^c^ External tools integrated with Blender to generate diverse crowd models. ^d^ HDRI (High Dynamic Range Imaging) is a 360° environment map that captures real-world lighting across a wide range of intensities.

**Table A2:** Detailed configuration of system layers in the scenario model.

Dataset	Layers	Functional Components	Software/Interface Used	Key Configuration	Outputs/Data Flow
ADSceneSim	L01	Physical dimensions	Pro-SiVIC^TM^ Actor Library:SivicCar, SivicPedestrain	Geometry follows the real-world specifications of actors(passenger car, van, truck, pedestrian)	3D actors registered in simulation scene
		Dynamic configuration	Actors Dynamics APIs	1. Physical dynamic properties: mass and inertia for vehicles; mass and collision radius for pedestrians 2. Motion parameters: vehicle suspension/tire dynamics; pedestrian gait parameters 3. Dynamic limits: vehicle speed, acceleration, and steering limits; pedestrian speed limits	Dynamic parameters registered for movable actors
	L02	Sensor types	1. Pro-SiVIC^TM^ Camera Library 2. RTMaps^TM^ Image I/O Blocks	1. Two RGB cameras (front and rear) mounted on the ego vehicle (Renault Zoe) 2. Connection between camera views and custom image I/O component in RTMaps^TM^	RGB cameras registered in simulation scene
		Sensor parameters	SivicCamera APIs	1. Camera mounting positions: front (1.88 m, 0, 1.15 m), rear (−0.22 m, 0, 1.15 m) 2. Front camera aligned with ego heading; rear rotated 180° around Y-axis 3. Field of View (FOV) 90°, resolution 1280 × 720, frame rate 25 Hz	Configured scripts defining camera setup and rendering properties
	L03	Event Execution	1. Pro-SiVIC^TM^ Event Library 2. RTMaps^TM^ Condition & Compute modules	1. Entity activation/deactivation, weather transitions, and time-scheduled actions 2. Conditional event parameters (bus-station Region of Interest (ROI) distance, waiting time) and actuator mappings (brake, steer, longitudinal gains)	Event triggers, conditional updates, and control feedback exchanged between Pro-SiVIC^TM^ and RTMaps^TM^
		Control Execution	1. Actors Dynamics APIs 2. RTMaps^TM^ Compute module 3. RTMaps^TM^ Sensor interfaces	1. Trajectory files (private .trk format) 2. Co-Pilot module integrated in RTMaps^TM^ [83,84,85] 3. HITL control via RTMaps^TM^ interfaces (keyboard/joystick to actuators)	Actors follow predefined trajectories or HITL/Co-Pilot commands
		Interface & recording	1. SivicObserver 2. RTMaps^TM^ Image I/O Blocks 3. SivicDDS/SivicFIFO	1. Observed actor state vectors 2. Camera reading/recording interfaces in Pro-SiVIC^TM^ 3. Data-sharing interfaces and synchronization mechanisms between Pro-SiVIC^TM^ and RTMaps^TM^	Real-time data exchange and synchronization; recording of sensor streams and scenario logs

## References

[1] (2021). Taxonomy and Definitions for Terms Related to Driving Automation Systems for On-Road Motor Vehicles.

[2] Kalra N., Paddock S.M. (2016). Driving to Safety: How Many Miles of Driving Would It Take to Demonstrate Autonomous Vehicle Reliability?.

[3] Khan A., Sohail A., Zahoora U., Qureshi A.S. (2020). A survey of the recent architectures of deep convolutional neural networks. Artif. Intell. Rev..

[4] Loh Y.P., Chan C.S. (2019). Getting to know low-light images with the exclusively dark dataset. Comput. Vis. Image Underst..

[5] Pitropov M., Garcia D.E., Rebello J., Smart M., Wang C., Czarnecki K., Waslander S. (2021). Canadian adverse driving conditions dataset. Int. J. Robot. Res..

[6] Shaik F.A., Reddy A., Billa N.R., Chaudhary K., Manchanda S., Varma G. Idd-aw: A benchmark for safe and robust segmentation of drive scenes in unstructured traffic and adverse weather. Proceedings of the IEEE/CVF Winter Conference on Applications of Computer Vision.

[7] Ros G., Sellart L., Materzynska J., Vazquez D., Lopez A.M. The synthia dataset: A large collection of synthetic images for semantic segmentation of urban scenes. Proceedings of the IEEE Conference on Computer Vision and Pattern Recognition.

[8] Gaidon A., Wang Q., Cabon Y., Vig E. Virtual worlds as proxy for multi-object tracking analysis. Proceedings of the IEEE Conference on Computer Vision and Pattern Recognition.

[9] Cabon Y., Murray N., Humenberger M. (2020). Virtual kitti 2. arXiv.

[10] Sun T., Segu M., Postels J., Wang Y., Van Gool L., Schiele B., Tombari F., Yu F. SHIFT: A synthetic driving dataset for continuous multi-task domain adaptation. Proceedings of the IEEE/CVF Conference on Computer Vision and Pattern Recognition.

[11] Gómez J.L., Silva M., Seoane A., Borrás A., Noriega M., Ros G., Iglesias-Guitian J.A., López A.M. (2025). All for one, and one for all: Urbansyn dataset, the third musketeer of synthetic driving scenes. Neurocomputing.

[12] HEADSTART Consortium (2019). HEADSTART: Harmonised European Solutions for Testing Automated Road Transport. https://headstart-project.eu/.

[13] Hi-Drive Consortium (2021). Hi-Drive: Advancing Short- and Long-Distance High-Level Automated Driving. https://hi-drive.eu/.

[14] AUGMENTED CCAM Consortium (2022). AUGMENTED CCAM: Augmenting and Evaluating the Physical–Digital Infrastructure for CCAM Deployment. https://augmentedccam.com/.

[15] SUNRISE Consortium (2021). SUNRISE: Safety, User Acceptance, and Interoperability in Automated Driving. https://sunrise-project.eu/.

[16] Esenturk E., Khastgir S., Wallace A., Jennings P. (2021). Analyzing real-world accidents for test scenario generation for automated vehicles. Proceedings of the 2021 IEEE Intelligent Vehicles Symposium (IV).

[17] De Gelder E., Paardekooper J.P., Saberi A.K., Elrofai H., Den Camp O.O., Kraines S., Ploeg J., De Schutter B. (2022). Towards an ontology for scenario definition for the assessment of automated vehicles: An object-oriented framework. IEEE Trans. Intell. Veh..

[18] Tarel J.P., Hautiere N., Cord A., Gruyer D., Halmaoui H. (2010). Improved visibility of road scene images under heterogeneous fog. Proceedings of the 2010 IEEE Intelligent Vehicles Symposium.

[19] Saleh F.S., Aliakbarian M.S., Salzmann M., Petersson L., Alvarez J.M. Effective use of synthetic data for urban scene semantic segmentation. Proceedings of the European Conference on Computer Vision (ECCV).

[20] Richter S.R., Vineet V., Roth S., Koltun V. (2016). Playing for data: Ground truth from computer games. Proceedings of the European Conference on Computer Vision.

[21] Alberti E., Tavera A., Masone C., Caputo B. (2020). IDDA: A large-scale multi-domain dataset for autonomous driving. IEEE Robot. Autom. Lett..

[22] Kloukiniotis A., Papandreou A., Anagnostopoulos C., Lalos A., Kapsalas P., Nguyen D.V., Moustakas K. CarlaScenes: A synthetic dataset for odometry in autonomous driving. Proceedings of the IEEE/CVF Conference on Computer Vision and Pattern Recognition.

[23] Sekkat A.R., Mohan R., Sawade O., Matthes E., Valada A. (2024). Amodalsynthdrive: A synthetic amodal perception dataset for autonomous driving. IEEE Robot. Autom. Lett..

[24] Geiger A., Lenz P., Urtasun R. (2012). Are we ready for autonomous driving? the kitti vision benchmark suite. Proceedings of the 2012 IEEE Conference on Computer Vision and Pattern Recognition.

[25] Karvat M., Givigi S. (2024). Adver-city: Open-source multi-modal dataset for collaborative perception under adverse weather conditions. arXiv.

[26] Wrenninge M., Unger J. (2018). Synscapes: A photorealistic synthetic dataset for street scene parsing. arXiv.

[27] Jadon A., Wang H., Thomas P., Stanley M., Cibik S.N., Laurat R., Maher O., Hoyer L., Unal O., Dai D. (2025). RealDriveSim: A Realistic Multi-Modal Multi-Task Synthetic Dataset for Autonomous Driving. arXiv.

[28] Wang X., Zhu Z., Huang G., Chen X., Zhu J., Lu J. (2024). Drivedreamer: Towards real-world-drive world models for autonomous driving. Proceedings of the European Conference on Computer Vision.

[29] Gao S., Yang J., Chen L., Chitta K., Qiu Y., Geiger A., Zhang J., Li H. (2024). Vista: A generalizable driving world model with high fidelity and versatile controllability. Adv. Neural Inf. Process. Syst..

[30] Wang Y., He J., Fan L., Li H., Chen Y., Zhang Z. Driving into the future: Multiview visual forecasting and planning with world model for autonomous driving. Proceedings of the IEEE/CVF Conference on Computer Vision and Pattern Recognition.

[31] Hu A., Russell L., Yeo H., Murez Z., Fedoseev G., Kendall A., Shotton J., Corrado G. (2023). Gaia-1: A generative world model for autonomous driving. arXiv.

[32] Ulbrich S., Menzel T., Reschka A., Schuldt F., Maurer M. (2015). Defining and substantiating the terms scene, situation, and scenario for automated driving. Proceedings of the 2015 IEEE 18th International Conference on Intelligent Transportation Systems.

[33] Menzel T., Bagschik G., Maurer M. (2018). Scenarios for development, test and validation of automated vehicles. Proceedings of the 2018 IEEE Intelligent Vehicles Symposium (IV).

[34] Winner H., Lemmer K., Form T., Mazzega J. (2019). PEGASUS—First steps for the safe introduction of automated driving. Proceedings of the Road Vehicle Automation 5.

[35] ASAM e.V. (2024). OpenDRIVE V1.8.1. https://www.asam.net/standards/detail/opendrive/.

[36] ASAM e.V. (2022). OpenSCENARIO V2.0.0. https://www.asam.net/standards/detail/openscenario/.

[37] ASAM e.V. (2021). OpenLABEL V1.0.0. https://www.asam.net/standards/detail/openlabel/.

[38] Dosovitskiy A., Ros G., Codevilla F., Lopez A., Koltun V. (2017). CARLA: An open urban driving simulator. Proceedings of the Conference on Robot Learning.

[39] Rong G., Shin B.H., Tabatabaee H., Lu Q., Lemke S., Možeiko M., Boise E., Uhm G., Gerow M., Mehta S. (2020). Lgsvl simulator: A high fidelity simulator for autonomous driving. Proceedings of the 2020 IEEE 23rd International Conference on Intelligent Transportation Systems (ITSC).

[40] Sippl C., Bock F., Lauer C., Heinz A., Neumayer T., German R. (2019). Scenario-based systems engineering: An approach towards automated driving function development. Proceedings of the 2019 IEEE International Systems Conference (SysCon).

[41] Zhang X., Tao J., Tan K., Törngren M., Sánchez J.M.G., Ramli M.R., Tao X., Gyllenhammar M., Wotawa F., Mohan N. (2022). Finding critical scenarios for automated driving systems: A systematic mapping study. IEEE Trans. Softw. Eng..

[42] Zhang X., Khastgir S., Tiele J.K., Takenaka K., Hayakawa T., Jennings P. (2024). Odd and behavior based scenario generation for automated driving systems. IEEE Access.

[43] Jeon J., Yoo J., Oh T., Yoo J. (2025). Simulation-Based Logical Scenario Generation and Analysis Methodology for Evaluation of Autonomous Driving Systems. IEEE Access.

[44] ASAM e.V. (2025). OpenODD V1.0.0. https://www.asam.net/standards/detail/openodd/.

[45] Thorn E., Kimmel S.C., Chaka M. (2018). A Framework for Automated Driving System Testable Cases and Scenarios.

[46] Koné T.F., Bonjour E., Levrat E., Mayer F., Géronimi S. (2019). Safety demonstration of autonomous vehicles: A review and future research questions. Proceedings of the International Conference on Complex Systems Design & Management.

[47] Hoss M., Scholtes M., Eckstein L. (2022). A review of testing object-based environment perception for safe automated driving. Automot. Innov..

[48] Abrecht S., Gauerhof L., Gladisch C., Groh K., Heinzemann C., Woehrle M. (2021). Testing deep learning-based visual perception for automated driving. ACM Trans. Cyber-Phys. Syst. (TCPS).

[49] Xu W., Gruyer D., Ieng S.S. (2023). Generic Simulation Framework for Evaluation Process: Applied to AI-powered Visual Perception System in Autonomous Driving. Proceedings of the 2023 IEEE 26th International Conference on Intelligent Transportation Systems (ITSC).

[50] Gyllenhammar M., Johansson R., Warg F., Chen D., Heyn H.M., Sanfridson M., Söderberg J., Thorsén A., Ursing S. Towards an operational design domain that supports the safety argumentation of an automated driving system. Proceedings of the 10th European Congress on Embedded Real Time Systems (ERTS 2020).

[51] Mendiboure L., Benzagouta M.L., Gruyer D., Sylla T., Adedjouma M., Hedhli A. (2023). Operational design domain for automated driving systems: Taxonomy definition and application. Proceedings of the 2023 IEEE Intelligent Vehicles Symposium (IV).

[52] Ercan S., Gruyer D., Hedhli A., Ieng S.S., Mendiboure L. (2024). Enhanced Taxonomy with Physical-Digital-Infrastructure (PDI) for Extended ODD Definition Applied to Connected and Cooperative Automated Mobility (CCAM). Proceedings of the 2024 IEEE 27th International Conference on Intelligent Transportation Systems (ITSC).

[53] Gruyer D., Grapinet M., De Souza P. (2012). Modeling and validation of a new generic virtual optical sensor for ADAS prototyping. Proceedings of the 2012 IEEE Intelligent Vehicles Symposium.

[54] Northcutt C.G., Athalye A., Mueller J. (2021). Pervasive label errors in test sets destabilize machine learning benchmarks. arXiv.

[55] Wang Z., Bovik A.C., Sheikh H.R., Simoncelli E.P. (2004). Image quality assessment: From error visibility to structural similarity. IEEE Trans. Image Process..

[56] Canny J. (2009). A computational approach to edge detection. IEEE Trans. Pattern Anal. Mach. Intell..

[57] Sobel I., Feldman G. (1968). A 3x3 isotropic gradient operator for image processing. Talk at the Stanford Artificial Intelligence Project (SAIL).

[58] IPG Automotive GmbH (2024). CarMaker. https://www.ipg-automotive.com/en/products-solutions/software/carmaker/.

[59] Gruyer D., Regnier R., Durand G., Chaves C., Quintero K. (2024). Deliverables WP2 2.9: Proofs-of-Concept Final Report–Development of Platforms Meeting the Desired Objectives of Evaluating Means of Automated Mobility.

[60] Gruyer D., Hedhli A., Ercan S., Ieng S.S., Benmoussa M., Boubezoul M., Ammoun S., Quintero K., Orfila O. (2024). Deliverables 4.1–Augmented Implementation and Validation Framework and Experimental Plans.

[61] Yu F., Chen H., Wang X., Xian W., Chen Y., Liu F., Madhavan V., Darrell T. (2020). BDD100K: A Diverse Driving Dataset for Heterogeneous Multitask Learning. Proceedings of the IEEE Conference on Computer Vision and Pattern Recognition (CVPR).

[62] Deschaud J.E. (2021). KITTI-CARLA: A KITTI-like dataset generated by CARLA Simulator. arXiv.

[63] Cordts M., Omran M., Ramos S., Rehfeld T., Enzweiler M., Benenson R., Franke U., Roth S., Schiele B. (2016). The cityscapes dataset for semantic urban scene understanding. Proceedings of the IEEE Conference on Computer Vision and Pattern Recognition.

[64] Sakaridis C., Dai D., Van Gool L. (2021). ACDC: The adverse conditions dataset with correspondences for semantic driving scene understanding. Proceedings of the IEEE/CVF International Conference on Computer Vision.

[65] Jocher G., Qiu J., Chaurasia A. (2023). Ultralytics YOLO, Version 8.0.0. https://github.com/ultralytics/ultralytics.

[66] Chen K., Wang J., Pang J., Cao Y., Xiong Y., Li X., Sun S., Feng W., Liu Z., Xu J. (2019). MMDetection: Open MMLab Detection Toolbox and Benchmark. arXiv.

[67] Contributors M. (2020). MMSegmentation: OpenMMLab Semantic Segmentation Toolbox and Benchmark. https://github.com/open-mmlab/mmsegmentation.

[68] Duminil A., Ieng S.S., Gruyer D. (2024). A comprehensive exploration of fidelity quantification in computer-generated images. Sensors.

[69] Duminil A., Ieng S.S., Gruyer D. (2025). Fidelity assessment of synthetic images with multi-criteria combination under adverse weather conditions. Sci. Rep..

[70] Haralick R.M., Shanmugam K., Dinstein I.H. (1973). Textural features for image classification. IEEE Trans. Syst. Man Cybern..

[71] Ojala T., Pietikainen M., Harwood D. (1994). Performance evaluation of texture measures with classification based on Kullback discrimination of distributions. Proceedings of the 12th International Conference on Pattern Recognition.

[72] Ahmed N., Natarajan T., Rao K.R. (2006). Discrete cosine transform. IEEE Trans. Comput..

[73] Ren S., He K., Girshick R., Sun J. (2015). Faster r-cnn: Towards real-time object detection with region proposal networks. Adv. Neural Inf. Process. Syst..

[74] Tian Z., Shen C., Chen H., He T. (2020). FCOS: A simple and strong anchor-free object detector. IEEE Trans. Pattern Anal. Mach. Intell..

[75] He K., Gkioxari G., Dollár P., Girshick R. (2017). Mask r-cnn. Proceedings of the IEEE International Conference on Computer Vision.

[76] Cao Y., Xu J., Lin S., Wei F., Hu H. (2019). Gcnet: Non-local networks meet squeeze-excitation networks and beyond. Proceedings of the IEEE/CVF International Conference on Computer Vision Workshops.

[77] Chen L.C., Zhu Y., Papandreou G., Schroff F., Adam H. Encoder-decoder with atrous separable convolution for semantic image segmentation. Proceedings of the European Conference on Computer Vision (ECCV).

[78] Long J., Shelhamer E., Darrell T. (2015). Fully convolutional networks for semantic segmentation. Proceedings of the IEEE Conference on Computer Vision and Pattern Recognition.

[79] Liu J., Zhan J., Zhang J., Chen J., Song Y., Tang L., Zhou L., Du C., Wei Y., Guo Y. (2025). Robust scale fusion and edge-aware feature attention network for remote sensing UAV road detection under harsh weather. Results Eng..

[80] Chen L.C., Papandreou G., Schroff F., Adam H. (2017). Rethinking atrous convolution for semantic image segmentation. arXiv.

[81] Hiblot N., Gruyer D., Barreiro J.S., Monnier B. Pro-sivic and roads. a software suite for sensors simulation and virtual prototyping of adas. Proceedings of the Driving Simulation-Conference Europe.

[82] Marc R., Dominique G., Evangeline P. (2012). Generator of road marking textures and associated ground truth applied to the evaluation of road marking detection. Proceedings of the 2012 15th International IEEE Conference on Intelligent Transportation Systems.

[83] Vanholme B., Gruyer D., Lusetti B., Glaser S., Mammar S. (2012). Highly automated driving on highways based on legal safety. IEEE Trans. Intell. Transp. Syst..

[84] Xu W., Sainct R., Gruyer D., Orfila O. (2021). Safe vehicle trajectory planning in an autonomous decision support framework for emergency situations. Appl. Sci..

[85] Xu W., Sainct R., Gruyer D., Orfila O. (2021). A decision support framework for autonomous driving in normal and emergencysituations. Proceedings of the 2021 AEIT International Conference on Electrical and Electronic Technologies for Automotive (AEIT AUTOMOTIVE).

